# Automated assessment of redox potentials for dyes in dye-sensitized photoelectrochemical cells†

**DOI:** 10.1039/d1cp04218a

**Published:** 2021-12-01

**Authors:** Jelena Belić, Arno Förster, Jan Paul Menzel, Francesco Buda, Lucas Visscher

**Affiliations:** Department of Chemistry and Pharmaceutical Sciences, Vrije Universiteit Amsterdam De Boelelaan 1083 1081 HV Amsterdam The Netherlands l.visscher@vu.nl; Leiden Institute of Chemistry, Leiden University Einsteinweg 55, P.O. Box 9502 2300 RA Leiden The Netherlands

## Abstract

Sustainable solutions for hydrogen production, such as dye-sensitized photoelectrochemical cells (DS-PEC), rely on the fundamental properties of its components whose modularity allows for their separate investigation. In this work, we design and execute a high-throughput scheme to tune the ground state oxidation potential (GSOP) of perylene-type dyes by functionalizing them with different ligands. This allows us to identify promising candidates which can then be used to improve the cell's efficiency. First, we investigate the accuracy of different theoretical approaches by benchmarking them against experimentally determined GSOPs. We test different methods to calculate the vertical oxidation potential, including *GW* with different levels of self-consistency, Kohn–Sham (KS) orbital energies and total energy differences. We find that there is little difference in the performance of these methods. However, we show that it is crucial to take into account solvent effects as well as the structural relaxation of the dye after oxidation. Other thermodynamic contributions are negligible. Based on this benchmark, we decide on an optimal strategy, balancing computational cost and accuracy, to screen more than 1000 dyes and identify promising candidates which could be used to construct more robust DS-PECs.

## Abstract 2

The alignment of the GSOP, calculated with the adiabatic approach for large number of dyes, with the limitations of a hypothetical system – the CB edge of TiO_2_ semiconductor and highest oxidation potential of Ru-based WOC.

## Graphical abstract

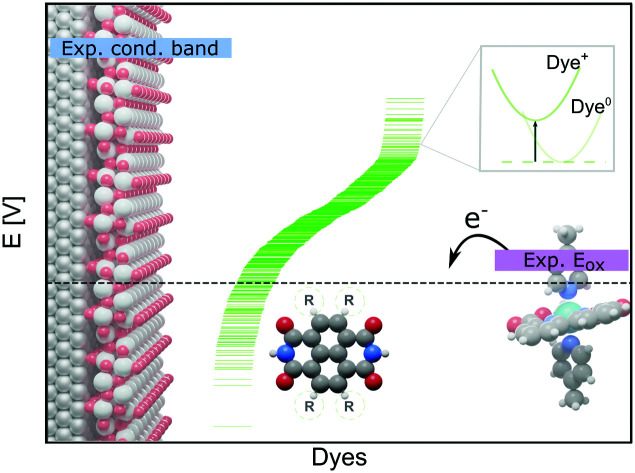

## 1 Introduction

Hydrogen is seen as a symbol of sustainable energy source, even though its production currently mostly relies on fossil fuels.^1,2^ To make production of this energy carrier more sustainable,^3–5^ a promising route is to split water with a dye-sensitized photoelectrochemical cell (DS-PEC) driven by solar energy.^6,7^ Such developments capitalize on the advantages of dye-sensitization, in particular the possibility to tune the dye's properties by small structural adjustments.^7^

**Fig. 1:**
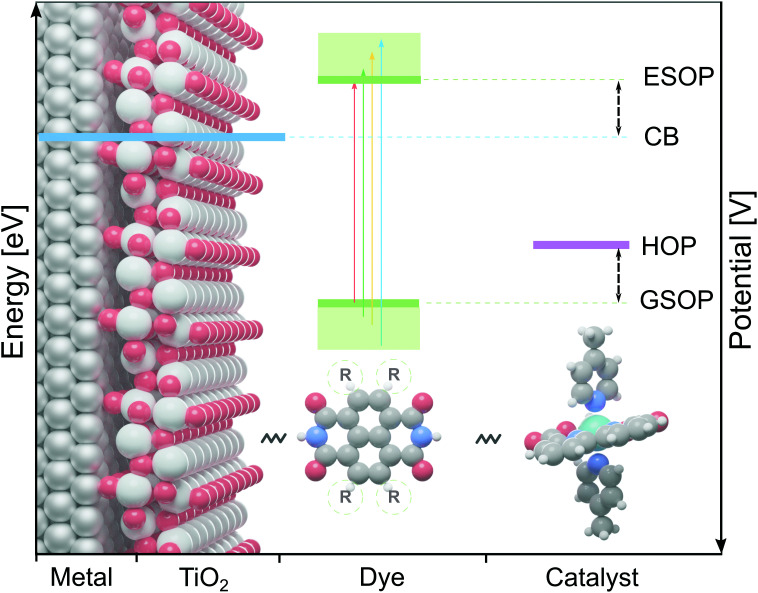
The alignment between redox potentials of the DS-PEC's components: conduction band (CB) edge of the anode (blue), ground state oxidation potential (GSOP) and excited state oxidation potential (ESOP) of the dye (green) and highest oxidation potential (HOP) of the water oxidation catalyst (purple).

In the present work we will focus on the dye on the photoanode of DS-PECs, for which the absorbed sunlight creates a photo-excited electron that is injected into the photo anode (usually TiO_2_). The thus oxidized dye is restored to its initial state by accepting an electron from a water oxidation catalysts (WOC), which then starts the water oxidation cycle.^7^ To make this process efficient the dye has to fulfill several requirements. Prerequisite for the chain of electron transfers (from the dye to the semiconductor and from the WOC to the dye) to proceed is a suitable alignment of the dye's redox potentials with the neighbouring components: (1) the ground state oxidation potential (GSOP) has to be higher than the highest oxidation potential (HOP) of the WOC and (2) the excited state oxidation potential (ESOP) has to be lower than the edge of the anode's conduction band (CB) (Fig. 1). While these can be considered as minimal requirements, one may further narrow the search for optimal dyes by estimating that for reducing the energy loss and for an optimal rate of the electron transfer the potential of the ESOP state needs to be just slightly higher (∼0.3 eV)^8^ than the semiconductor's CB.

A further boundary condition for sustainable deployment of DS-PECs is the use of dyes made entirely from abundant material, such as fully organic dyes. Among these, perylene diimides (PDI) have been recognised as very promising for use in photovoltaic devices^7,9^ due to their favourable optical and electrochemical properties, as well as their stability, relatively cheap synthesis and tunability. They have found a place in different types of photo-electrochemical devices, both at the anode^10–13^ and at the cathode.^14^ Also other dyes related to the perylene diimide have proven to be efficient components of such systems, like naphtalene diimide (NDI)^15^ or perylene triimides (PTI).^16^

Given this versatility of the perylene group of dyes, there are thousands of dyes that could be synthesized and tested for their performance. To better narrow down the search for optimal performers, the use of computational modelling is of interest. With a clear idea and well-defined objectives, such a computational screening of the chemical space can strongly reduce the amount of trial experiments. Important is thereby to take into account the uncertainties that the construction of model and the choice of theoretical methods bring. In a complex system there are many factors that affect the final performance and theory should merely narrow down the search rather than finding the perfect candidate. A great advantage is the modularity of the DS-PEC system, thus one may study its molecular components separately and define suitable components before constructing an overall model. Studies on molecular components therefore encompass dyes with favourable light absorption properties,^17^ anchoring groups between the dye and the semiconductor for ultrafast electron injection^18^ and the WOC–dye complex for driving the water splitting process.^19,20^ Analysis and screening of the individual components will help to define optimal DS-PECs and to improve upon the hydrogen production efficiency. In this work we focus on finding dyes that fit the system's thermodynamic requirements, in particular those that have a suitable GSOP.

The GSOP can be predicted by calculating the Gibbs free energy of the oxidation reaction,^21^ the difference between the solution-phase Gibbs free energies of a molecule in neutral and oxidized form. We can access these thermodynamic properties using generalized^22,23^ Kohn–Sham (KS)^24^ density functional theory (DFT).^25^ As reactions take place in a medium, solvent effects need to be accounted for which is most efficiently done with continuum solvation (CS) models.^26^ Furthermore we may distinguish between two major pathways that are commonly used to calculate solution-phase Gibbs free energy: use of a thermodynamic cycle (TC) and direct computation (DC) within a CS model.

Simpler than these two thermodynamical approaches is to approximate the GSOP by the vertical ionization energy of the solvated dye. Such a calculation can be done in three ways, one may compute the electronic energy difference by two separate KS-DFT calculations of the molecule in its two forms (before and after oxidation), or one may take the GSOP as the negative of the highest occupied molecular orbital (HOMO) energy of the neutral molecule. For the latter approach, it is important to take into account that semi-local or hybrid approximations to the exact functional of DFT do not describe the physics of addition or removal of electrons fully correctly.^27–30^ A theoretically more rigorous alternative is many-body perturbation theory (MBPT) in the *GW* approximation (GWA) to the electronic self-energy,^31^ where *G* denotes the single-particle Green's function and *W* is the screened Coulomb interaction. As in approaches based on total energy differences, the GWA accounts for the systems response to the hole created by ionization.^32^ It yields QP energies which can be directly identified with vertical electron addition and removal energies^33^ and is therefore increasingly used for energy level alignment in photo-catalytic interfaces^34,35^ or dye-sensitized solar cells.^36–39^

The most commonly used way to validate theoretical predictions of the GSOP is a comparison to the oxidation potential measured by cyclic voltammetry (CV). The half-reaction (Dye → Dye^+^ + e_anode_^−^) taking place at the surface of the anode, with the dye redox couple [Dye/Dye^+^] rapidly exchanging electrons with the electrode, is thereby considered to be electrochemically reversible. The measured half-wave potential *E*_1/2_ of the reaction is, at standard conditions, a very good estimate for the standard electrode potential: *E*_1/2_ ≈ *E*°,^40^ with *E*° being the driving force for electrochemical work.

As we are primarily interested in the GSOP here, the measured *E*_1/2_ potentials can directly be used as experimental reference data to validate the accuracies of the different methods to calculate the GSOPs. Treatment of the ESOP is a bit more involved as this depends on the GSOP as well as on the energy difference between the ground and excited state. The latter can be computed as the adiabatic energy difference between the ground state and the geometry-relaxed excited state, but also as a vertical excitation energy under the assumption that the electron transfer is much faster than the nuclear relaxation (Franck–Condon principle). Which picture is the most appropriate depends on the dye-semiconductor system; it has been observed for some semiconductors that the injection happens only after the relaxation.^8^ One may also note that for certain dyes the excited-state reorganisation energy is very small, making the differences between the schemes less important.^41^

In this work we aim to construct and validate a fast procedure for the calculation of GSOPs which can be used for the automated screening of the GSOPs for different sets of dyes and their derivatives. In this construction step we consider accuracy as well as computational efficiency and discuss the effect of different approximations on these factors. This work represents a step towards full automation for the screening of dyes for use in DS-PECs – an automated strategy to obtain accurate energetics of the system's interfaces.

## 2 Methods

In this section we will discuss the possible computational strategies for GSOP determination and the experimental data that can be used to validate them. We will start with the two adiabatic strategies directed towards full determination of the Gibbs free energy, consider in the second section approaches that use the vertical ionization potential, and end with the selection of the experimental reference data.

### 2.1 GSOP – adiabatic approach

In the adiabatic approach, the GSOP is defined as the absolute difference between the solution-phase Gibbs free energies of the products and reactants, Dye and Dye^+^ + e^−^. It describes the energy change between the molecular species in their respective solvation and thermal equilibrium conditions. The Gibbs free energy of the electron depends on the choice of the statistical mechanical formalism – which does not matter as long as it is consistent with the formalism of the reference electrode, which we will address later. For the Fermi–Dirac statistics this value is −3.62 kJ mol^−1^ (−0.0375 eV).^42,43^ Solution-phase Gibbs free energies *G*^*i*^_sol_(*g*^*i*^_sol_), where the index *i* labels the neutral (0) or oxidized (+) forms of the dye and *g* stands for the optimized geometry of the species, can be calculated using DFT. We thereby distinguish between the dye's energy *E*^*i*^_sol_(*g*^*i*^_sol_) and thermal contributions *G*^*i*^_therm_(*g*^*i*^_sol_,*T*) at the temperature *T* of interest:

1 *G*^*i*^_sol_(*g*^*i*^_sol_) = *E*^*i*^_sol_(*g*^*i*^_sol_) + *G*^*i*^_therm_(*g*^*i*^_sol_,*T*)

The subscripts hereby indicate the phase of the species: solvated (sol) or in gas phase.

The thermal contribution is the most demanding term in the calculation as it requires determination of the vibrational frequencies of the molecule. The *G*^*i*^_therm_(*g*^*i*^_sol_,*T*) is computed within an ideal gas model and includes, besides zero-point vibrations, the translational and rotational contributions to nuclear kinetic energy. In the computation of the entropy term the quasi-rigid-rotor-harmonic-oscillator approximation model^44^ for weak (less than 20 cm^−1^) vibrational modes is employed to avoid artifacts due to inaccuracies in these modes.

**Fig. 2:**
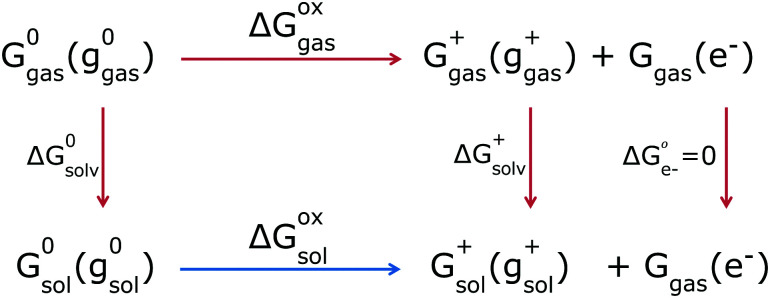
The thermodynamic cycle. The molecule's total Gibbs free energies are denoted with G and molecule's geometry with g in brackets; the subscript is the phase and in superscript is the oxidation state of the species. Solvation processes are the vertical and oxidation processes are the horizontal reactions. Next to the arrows are changes in Gibbs free energies for each process. The free electron is by electrochemical convention always in gas phase.

Given these individual Gibbs free energies, the GSOP can then be calculated as the difference (Δ*G*^ox^_sol_) between the solution-phase Gibbs free energies of a neutral and charged species (Fig. 2, bottom reaction, blue arrow). We will refer to this way of calculation as direct computation (DC). This procedure is rather straightforward and the final value of the adiabatic GSOP with DC and COSMO^45^ as CS model, Δ*G*^DC^_COSMO_ is calculated using eqn (2):

2 Δ*G*^DC^_COSMO_ = *G*_sol_^+^(*g*_sol_^+^) + *G*_gas_(e^−^) − *G*^0^_sol_(*g*^0^_sol_)

*G*_sol_^+^(*g*_sol_^+^) and *G*^0^_sol_(*g*^0^_sol_) are solution-phase Gibbs free energies of the oxidised and neutral molecular species, respectively.

An alternative is the use of a thermodynamic cycle (TC) method (Fig. 2, top reactions, red arrows). In the TC method, a gas-phase Gibbs free energy is augmented by solvation contributions to yield a solution-phase Gibbs free energy. For each oxidation state (neutral, 0, or ionized, +) three separate contributions are computed: the gas-phase Gibbs free energy *G*^*i*^_gas_(*g*^*i*^_gas_) (following eqn (1) for gas phase), the Gibbs free energy of solvation computed at the gas phase geometry *g*^*i*^_gas_, and the energy difference between the solvated and gas phase structure:

3 *G*^*i*^_sol_(*g*^*i*^_gas_) = *G*^*i*^_gas_(*g*^*i*^_gas_) + Δ*G*^*i*^_solv_(*g*^*i*^_gas_) + [*E*_gas_(*g*^*i*^_sol_) − *E*_gas_(*g*^*i*^_gas_)]

The equation for the adiabatic GSOP computed with the TC method and COSMO, Δ*G*^TC^_COSMO_ is then similar to eqn (2) and reads:

4 Δ*G*^TC^_COSMO_ = *G*_sol_^+^(*g*_gas_^+^) + *G*_gas_(e^−^) − *G*^0^_sol_(*g*^0^_gas_)

The DC and TC strategies can be expected to give close agreement if the structure and vibrational frequencies of the dye are only weakly affected by solvation. If the stationary points in solution strongly differ from the ones in the gas phase, the DC and TC approach will deviate from each other.^46,47^ For the thermal correction the use of a thermodynamic cycle is then often considered to be more robust^48^ as CS models are parametrised to yield accurate Gibbs free energies of solvation (Fig. 2, vertical red lines).^46,49–52^ This implies that the thermal contributions calculated in a dielectric continuum in the (DC strategy) can contain corrections that are already incorporated in the CS model (*via* parametrization) which might result in double counting for some solvent effects. Ho^47^ compared the thermal contributions (particularly vibrational corrections) obtained by the two strategies, TC and DC, and showed that mean absolute errors in the redox potential for the two strategies differ by 10 mV from each other. In this work he also considered introducing a higher-level electronic structure theory, but found that this did not necessarily lead to better agreement with experiment, indicating that the description of solvation *via* a CS model is the main source of errors. Regarding the choice between the DC and TC approach, we furthermore note that in the TC approach evaluation of multiple solvents is more economical as the most demanding step of the calculation, determination of vibrational frequencies, is independent of the chosen solvent. If only one solvent is to be considered, the TC and DC strategies are similar in terms of computational costs. In DC we avoid the separate calculations for the gas and solution phase that are needed in the TC approach, but the effort needed for frequency calculations in solution is typically higher than in gas phase.

As a cheaper alternative in the TC pathway we can also employ the conductor-like screening model for realistic solvents (COSMO-RS),^53,54^ which performs statistical thermodynamics for the molecular surface interactions after the quantum chemical calculation. While COSMO provides the Gibbs free energy of solvation only (vertical red reaction), COSMO-RS provides the chemical potential of a solvated molecule and allows calculating all fluid phase equilibrium thermodynamic properties which includes the solution-phase Gibbs free energy as well.^50,55^ The low cost of this model lies in statistical thermodynamics part whose cost is negligible compared to quantum chemical calculations and which makes the explicit calculation of thermal contributions redundant, as it directly provides the solution-phase Gibbs free energy. The solution-phase Gibbs free energy thus reduces to a sum of the gas-phase energy and a COSMO-RS Gibbs free energy correction. The geometry changes due to solvation are ignored,

5 *G*^*i*^_sol,CRS_(*g*^*i*^_gas_) = *E*^*i*^_gas_(*g*^*i*^_gas_) + Δ*G*^*i*^_CRS,solv_(*g*^*i*^_gas_)

Inserting the solution-phase Gibbs free energy defined in eqn (5) into eqn (4), the adiabatic GSOP using the TC pathway in combination with COSMO-RS is obtained.

Finally, to be able to compute a large number of molecules, we can reduce the time spent for geometry optimization. A workflow combining semiempirical quantum mechanical (SQM) techniques for geometry optimisations and frequency calculations and DFT electronic energy with COSMO is shown to provide reliable predictions of redox potentials.^56,57^ Similarly, we can employ the SQM method instead of DFT for geometry optimisation and combine it with DFT electronic energies and the COSMO-RS solvation model. The combination of SQM methods with a COSMO-RS thermodynamic calculation is very efficient and the limiting step in this workflow will be the single point DFT calculation including COSMO. A dedicated COSMO calculation is needed to define the molecule's reference state that is used in further COSMO-RS statistical thermodynamics calculations. As COSMO-RS provides the solution-phase Gibbs free energy, no frequency calculations are needed. We will denote the result of this composite method as Δ*G*^screening^_COSMO-RS_.

### 2.2 GSOP – vertical approach

The Gibbs free energy difference should correspond exactly to the GSOP measured in the CV experiments as the time scale of these experiments is long enough to allow for full relaxation. Computing the GSOP in the vertical approximation and neglecting vibrational effects is computationally attractive and can be considered a valid approximation if the system is not in thermodynamical equilibrium, which will be the case when the electron transfer process is fast enough. Physically, the relaxation of the neutral geometry after electron removal is then neglected and only relaxation of the electrons as a response to the removal of an electron to the system is accounted for ref. 32. In this regime one can consider three different strategies.

#### 2.2.1 Calculation as total electronic energy difference

The first approach is to compute total electronic energy for both the neutral and oxidized species at the optimized geometry of neutral molecule in solvent. The total energy difference

6 Δ*E*^ox^ = *E*_sol_^+^(*g*^0^_sol_) − *E*^0^_sol_(*g*^0^_sol_)

is then taken as approximation to the GSOP.

#### 2.2.2 Calculation as KS-DFT HOMO energy

The simplest approach would be to identify the negative of the HOMO energy, −*ε*_HOMO_, from a KS-DFT calculation with the vertical ionisation energy in exact KS-DFT. While for any finite system −*ε*_HOMO_ exactly equals the ionisation energy in exact KS-DFT,^58^ errors obtained from common approximations to the exact exchange–correlation potential *v*_xc_ can be of the order of several eV.^29,30,59^ This failure is a direct consequence of the incorrect long-range behaviour of the *v*_xc_(*r*) (exponentially instead of 1/|*r*|) in the local density approximation (LDA) and generalized gradient approximations (GGA).^28^ On the other hand, the exact exchange (eex) potential shows the correct long-range behaviour^60^ and hybrid functionals, combining GGAs with a fraction of eex give typically much better ionisation energies. Despite these obvious shortcomings, the KS-DFT approach is computationally very efficient since it only requires to perform a single geometry optimization in solution.

#### 2.2.3 Calculation as *GW* HOMO energy

In a single-particle energy framework, one can also go beyond DFT and calculate a vertical approximation to GSOP using MBPT. Central to MBPT is Dyson's equation^61^7
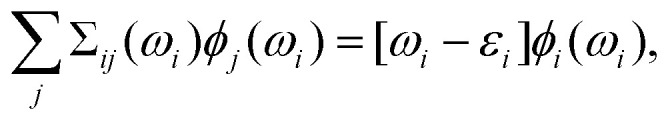
where *ε*_*i*_ is a KS eigenvalue, *ϕ*_*i*_ denotes a molecular orbital, *ω*_*i*_ is the exact one-electron energy and Σ is the so-called self-energy. In practice, the GWA to the self-energy^31^ is often used. In the GWA, Σ is calculated as the convolution (in frequency space) of the interacting Green's function *G* and the dynamically screened interaction *W*, which is related to the unscreened Coulomb interaction *v*_c_ by

8 *W*_0_(*ω*) = [*ε*_RPA_(*ω*)]^−1^*v*_c_, *ε*_RPA_ = 1 + *v*_c_π_0_(*ω*),

where π_0_ is the independent particle polarizability in the RPA. Eqn (7) is usually simplified by evaluating Σ with a non-interacting Green's function, *G*_0_, instead of the interacting one. In the *G*_0_*W*_0_ approximation^62^ Σ is additionally approximated as diagonal, so that the eqn (7) reduces to a set of independent non-linear equations,

9 Σ_*ii*_(*ω*_*i*_) = *ω*_*i*_ − *ε*_*i*_,

with Σ_*ii*_ = *ϕ*_*i*_|Σ(*ω*)|*ϕ*_*i*_. Thus, the QP energies are obtained as a perturbative correction to the KS eigenvalues. In eigenvalue-only self-consistent *GW* (ev*GW*), the QP energies are additionally updated until self-consistency is reached.^62^ Yet another option is to map Σ to a static, non-local and Hermitian exchange–correlation potential,^63,64^ which then defines a non-linear eigenproblem, much like in KS-DFT, with the only difference that the potential is a functional of *G*_0_. This approach is referred to as QP self-consistent *GW* (qs*GW*). The *GW* approximation can be implemented with almost quadratic scaling using localized atomic orbitals,^65–67^ which makes at least *G*_0_*W*_0_ competitive with hybrid functional calculations in terms of computational cost in the same basis set.^67^ Self-consistent variants are more expensive, but the calculations typically converge in 5–10 iterations.^68^ However, *GW* calculations converge much slower to the complete basis set (CBS) limit.^33,69,70^ For accurate QP energies, it is usually necessary to perform calculations using correlation consistent basis sets of at least triple- and quadruple-*ζ* quality and to extrapolate to the CBS limit.^69–71^

### 2.3 Experimental data set

**Fig. 3:**
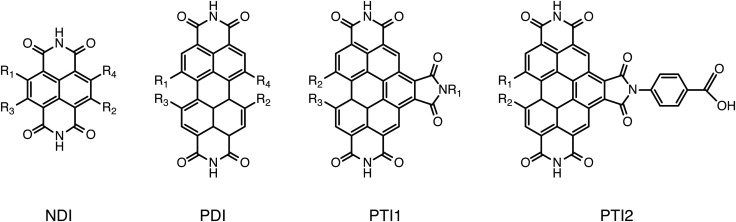
Structures of the unsubstituted cores considered for screening.

To validate the methods discussed above we compared the calculated GSOP values with experimental values for *E*_1/2_. The set used for validation is a subset of all considered dye cores (Fig. 3) for which we only selected those GSOP measurements that were done under similar experimental conditions. The substituted naphthalene-diimides (NDI),^72–75^ perylene-diimide (PDI)^76^ core and substituted PDI cores^76,77^ with different number and different type of substituents are used for validation of theoretical predictions.

**Fig. 4:**
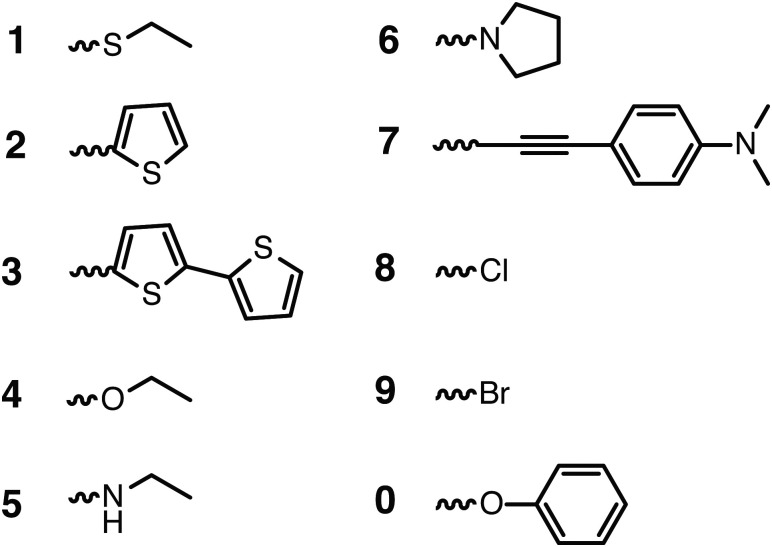
Structures of the substituents attached to the cores.

Each substituent is given a single character for identification to shorten the notation for the complete dye in which the substituents are attached to the aromatic core, as shown in Fig. 4. The substituents containing electron donating atoms and groups are numbered from 1 to 7. The substituents 8, 9 and 0 are introduced only to enlarge the experimental data set available for validation and they are not used in the final screening procedure. As shown in Fig. 4, side chains of the substituents and cores were sometimes shortened for computational convenience. We thereby checked that the consequence of such replacements on the computed GSOPs are minor, and moreover, since this is done systematically for all the dyes this should give at most a consistent shift in oxidation potential value (ESI,† S1). For comparison of electrochemical properties from different experiments or for comparison of computational results with experiment we need to convert all values to a common scale, the absolute scale (against vacuum). Choosing Fermi–Dirac statistics as statistical mechanical formalism for the electron we set the absolute potentials for *F*_c_/*F*_c_^+^ couple as 4.98 V and for SCE as 4.52 V (ESI,† S1).^43^ The complete set of dyes with experimental oxidation potentials can be found in the ESI,† (Table S1).

To quantify the predicting ability of the methods we will use mean deviations and squared correlation – *R*^2^. The magnitude of mean deviations mainly depends on the choice of the formalism to relate the reference electrode scale to the absolute scale. This choice might yield higher or lower mean deviations, but the shift will be consistent throughout the data set. For this reason we will rely on the *R*^2^ – strength of the linear correlation – for the evaluation of the performance. Having in mind possible errors from experimental and theoretical methods we monitored how sensitive *R*^2^ is to small variations in the reference values. Considering the errors that might occur as a consequence of side chain adjustments we tested how much *R*^2^ deviates from its initial value if we introduce a random error in the (+0.05, −0.05) eV interval. We found that the mean absolute deviation of the *R*^2^ converges to 0.01 (ESI,† S2). In conclusion, the methods for which the correlation with the experiment differs in ±0.01 would be considered to perform equally well.

## 3 Computational details

All computations were performed with the Amsterdam Modeling Suite (AMS) from Software for Chemistry and Materials (SCM),^78^ versions 2019.302 and 2020.1, except for the *GW* calculations which were performed with a modified development version of 2020.1. Initial geometries for the core and the library of substituents were prepared with the SCM Graphical User Interface (GUI). The long alkyl chains were replaced with ethyl groups. Generation of NDI and PDI derivative structures was done with the Compound Attachment Tool (CAT).^79^ CAT is a Python code employing, among others, the Python Library for Automating Molecular Simulation (PLAMS)^80^ for generating structures. The initial molecular structures were pre-optimized with the DFTB3^81^ method using the 3-ob parameter set.^82–85^ Convergence to a stable minimum of the nuclear potential energy surface was checked by calculating only the lowest frequency normal modes.^86^ We allowed for some numerical noise and considered structures with imaginary frequencies above −20 cm^−1^ for further characterisation. The workflow for the strategies above in general consists of geometry optimization (GO) in some cases followed by frequencies (normal modes calculation) and single point (SP) calculation(s). SP calculations were done with DFT or GW. Depending on the strategy, solvation effects were included in the geometry optimization calculation or in the following single point calculation (ESI,† S3). The DFT calculations (GO and SP) were done using the B3LYP functional,^87^ with the TZ2P basis set.^88^

The quality of density fitting and numerical integration were set to *Good*, except for the part with Hartree Fock exchange where quality was set to *Normal*. The SQM technique for GO in the screening workflow was GFN1-xTB.^89^ For the dyes that showed too large imaginary frequencies the GO calculation was repeated after tightening the convergence criteria for energy and nuclear gradients by a factor of 10 relative to the default values. The frequency calculations for hybrids were done numerically; their outputs contain all the thermodynamic properties, electronic bonding energy and nuclear kinetic energies, at room temperature. If present, negative frequencies were re-scanned to assure that they were not spurious (tolerating frequencies above −20 cm^−1^).

Solvation effects were incorporated with the COSMO^90^ or COSMO-RS^91^ models, at a pressure of 1 atm and temperature of 298.15 K. All the calculations including the COSMO model were performed in dichloromethane, with a dielectric constant of 8.9. The default Amsterdam density functional (ADF) 2019.302^92^ settings were used: the cavity construction is Delley type,^93^ atomic radii are the corresponding van der Waals radii from the MM3 method by Allinger^94^ (with widely accepted increase of 120%^54^). COSMO-RS requires an input from a COSMO calculation that defines the reference state which is the molecule in a perfect conductor, with infinite dielectric constant. In the reference COSMO calculation, the cavity construction is of Delley type and atomic radii are the Klamt atomic radii.^54^ The subsequent statistical thermodynamic calculation describes the effect of dichloromethane as a solvent.

The *GW* calculations were based on the solvated geometries optimized with B3LYP/TZ2P. We considered different variants of non self-consistent *GW* (*G*_0_*W*_0_) and partially self-consistent *GW*: *G*_0_*W*_0_@PBE0(HF = 0.4) (*G*_0_*W*_0_ performed on top of a PBE0^95,96^ calculations with 40% exact exchange), *G*_0_*W*_0_@LRC-*ω*PBEH,^97^ eigenvalue-only self-consistent *GW* (ev*GW*) based on the same two functionals (ev*GW*@PBE0(HF = 0.4) and ev*GW*@LRC-*ω*PBEH), QP self-consistent *GW* (qs *GW*) as well as QP self-consistent *GW* with self-consistency only in *G*, but not in *W* based on a PBE starting point (qsG*W*_0_@PBE). All *GW* calculations were performed using the TZ2P and TZ3P^71^ basis sets. For *G*_0_*W*_0_@PBE(HF = 0.4) we additionally used the QZ6P basis set^71^ and extrapolated the IP to the CBS limit using10
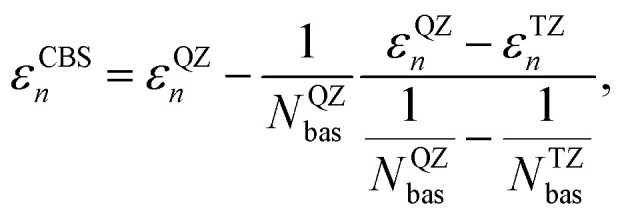
where *ε*^QZ^_*n*_ (*ε*^TZ^_*n*_) denotes the value of the QP energy using QZ6P (TZ3P) and *N*^QZ^_bas_ and *N*^TZ^_bas_ denote the respective numbers of basis functions (in spherical harmonics so that there are 5d and 7f functions).^71^

The *GW* implementation in ADF follows the space-time method and is outlined in ref. 67 and 71 as well as in ref. 68 for qs*GW*_0_ and qs*GW* and the numerical settings chosen for all the *GW* calculations in this work follow the recommendations therein: We used 20 grid points in imaginary time and imaginary frequency each. For the *G*_0_*W*_0_ and ev*GW* we set NumericalQuality *Good* and for the qs*GW*_0_ calculations we used *veryGood* quality combined with the *Good* fit sets and thresholds for the HF part of the gKS and for the *GW* part of the calculations. For reasons outlined in ref. 67 we set *Dependency Bas = 5 × 10*^*−4*^ in the AMS input for *G*_0_*W*_0_ and ev*GW* calculations and Dependency Bas = 5 × 10^−3^ for qs*GW*_0_.

## 4 Results

### 4.1 Adiabatic GSOP

**Fig. 5:**
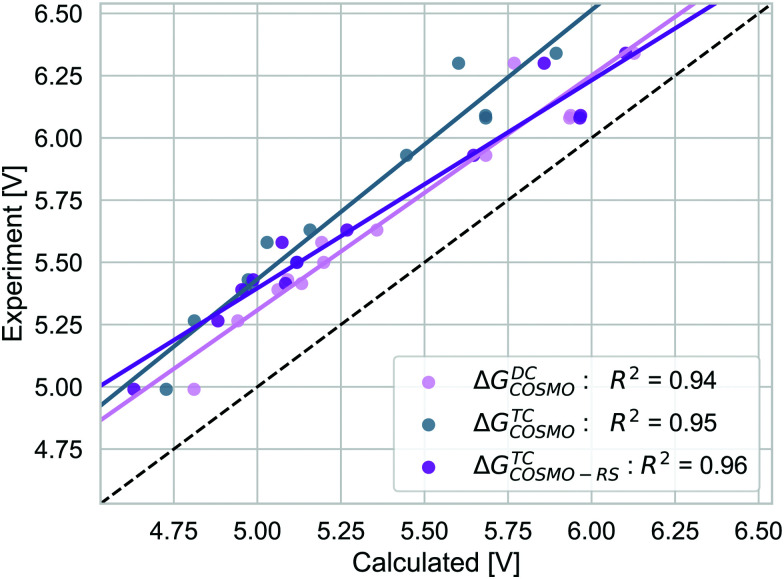
The correlation of adiabatic GSOP computed with Δ*G*^TC^_COSMO_ (pink), Δ*G*^DC^_COSMO_ (grey) and Δ*G*^TC^_COSMO-RS_ (purple) methods to the experimental oxidation potential *vs.* vacuum (dashed line).

We compare the strategies for obtaining the Gibbs free energy of the oxidation reaction with two pathways: TC and DC with COSMO. For the TC strategy we additionally consider the COSMO-RS method. The performance of these methods is determined for 13 out of 14 molecules from the initial data set. We excluded the PDI-0000 molecule as it turned out to heavily influence the correlation with the experimental reference data and thus presents a clear outlier. The unexpectedly large errors seen for this molecule may be caused by uncertainties in selecting its most appropriate conformation^98^ or by its sensitivity to CS model parameters (ESI,† S4). Both aspects make this molecule unsuited for getting accurate results with the automated procedure for initial structure generation and subsequent calculations that we currently employ. Table 1 shows that calculation timings are similar for the Δ*G*^TC^_COSMO_ and Δ*G*^DC^_COSMO_ method. Both methods perform equally well for the given set of experimental results, Fig. 5. The TC method has a slightly higher squared correlation coefficient (*R*^2^ = 0.95) than the DC methods (*R*^2^ = 0.94). The values from the TC method have an almost consistent shift from the corresponding experimental values, with a mean absolute deviation of 0.45 eV (RMSD = 0.46 eV). The DC method is a bit closer to the absolute experimental values with a MAD of 0.28 eV (RMSD = 0.30 eV). The Δ*G*^TC^_COSMO-RS_ absolute deviation of 0.34 eV (RMSD = 0.36 eV) is as large as for other two methods, while the squared correlation is higher than both methods employing only COSMO, *R*^2^ = 0.96. The Δ*G*^screening^_COSMO-RS_, where the geometry optimisation is performed using the SQM method (GFN1-xTB), performs as well as Δ*G*^TC^_COSMO-RS_. The correlation between these two methods is almost 1 and for both methods the correlation with experiment is 0.96.

**Table 1:** The strategies considered in this publication together with required calculations, different methods, and corresponding computation times. Method performance was accessed for DFT^c^ with B3LYP functional in combination with other methods. Timings denote elapsed times measured in CPU hours and are for unsubstituted PDI with 40 atoms

Approach	Calculations^a^	Method	Elapsed (step)^b^	Total (core h)^b^
Δ*G*^DC^_COSMO_	GO(n,s) + f	DFT	390	1066
	GO(o,s) + f		676	

Δ*G*^TC^_COSMO_	GO(n,v) + f	DFT	381	1109
	+SP(n,s)		1.4	
	GO(n,s)		9.6	
	+SP(n,v)		1.3	
	GO(o,v) + f		692	
	+SP(o,s)		2.5	
	+SP(n,v)		1.3	
	GO(o,s)		18.3	
	+SP(n,v)		1.3	

Δ*G*^TC^_COSMO-RS_	GO(n,v)	DFT	9.6	32.7
	+SP(n,s)		1.4	
	GO(o,v)		19.2	
	+SP(o,s)		2.5	

Δ*E*^DFT^_ox_	GO(n,s)	DFT	9.6	12.1
	+SP(o,s)		2.5	
*ε* ^DFT^_HOMO_	GO(n,s)	DFT	9.6	9.6
*ε* ^*GW*^_HOMO_	GO(n,s)	DFT	9.6	47.7
	+SP(o,s)	DFT	2.5	
	+SP(n,v)	*G*_0_ *W*_0_ d	29 + 6.6	

Δ*G*^screening^_COSMO-RS_	GO(n,v)	GFN-xTB	0.1	4.1
	+SP(n,s)	DFT	1.4	
	GO(o,v)	GFN-xTB	0.1	
	+SP(o,s)	DFT	2.5	

a GO denotes geometry optimization of the neutral (n) or oxidized (o) molecule in vacuum (v) or in solution (s); f denotes frequencies; SP denotes single point calculation using the preceding geometry. All geometry calculations are started from the same initial geometry.

b All calculations have been performed on a single 2.2 GHz intel Xeon (E5-2650 v4) node (broadwell architecture) with 24 cores and 128 GB RAM.

c The TZ2P basis set and B3LYP-D3(BJ) functional has been used for all DFT calculations. *Good* numerical quality has been used throughout except for the Hartree–Fock part, where the *Normal* fit set has been used.

d (QZ6P + TZ3P).

As the GSOP represents the difference between the two different oxidation states of the same molecule, some contributions will cancel out. Here we elaborate on the effective contributions to the GSOP originating from solvation, geometry relaxation due to oxidation and due to solvation and thermal contribution. The energy contribution due to geometry relaxation related to the solvation in the TC method is added separately from electronic solvation effects, as shown in eqn (3).

We found that this energy difference for both, neutral or oxidized molecules is negligible, being on average 0.02 eV and independent from type or number of ligands (ESI,† S5). Therefore, for this type of molecules solvated in dichloromethane the energy contribution coming from the change in geometry due to the solvation is negligible. In the TC method, this means that evaluation of these contributions can be omitted to reduce the total computational cost of the Δ*G*^TC^_COSMO_ strategy.

On the other hand, the electronic solvent contributions are crucial to the GSOP evaluation. The effective contribution to the GSOP is the difference between the solvation effects on neutral and oxidized molecules, eqn (11). For the TC method these values are calculated in the gas-phase. In the DC method this solvent contribution is contained in the *E*^*i*^_sol_(*g*^*i*^_sol_) energy term in eqn (1). On average the value of ΔΔ*G*_solv_ is −1.61 eV, with a maximum value of −2.00 eV for NDI-58. This contribution does not depend largely on the method, TC or DC (ESI,† S5)

11 ΔΔ*G*_solv_ = Δ*G*_solv_^+^ − Δ*G*^0^_solv_

We also evaluated the effective contribution of the thermal effects on the GSOP. Depending on the method, the thermal contribution is evaluated in solution or gas-phase for the DC or TC method respectively, for the temperature *T* = 298.15 K.

12 Δ*G*^DC(TC)^_therm_(*T*) = *G*_therm_^+^(*g*_sol/gas_^+^,*T*) − *G*^0^_therm_(*g*^0^_sol/gas_,*T*)

The effective Δ*G*^DC(TC)^_therm_(*T*) contribution for DC and TC, has an average of 0.04 eV and 0.03 eV respectively (for the absolute value difference). So, frequencies calculated in the same environment are very close in value for neutral and oxidized dyes and their contribution cancel out. Considering the cost of frequency calculations listed in Table 1, for GO(n,s) + f and just GO(n,s), the frequencies are 97% of GO(n,s) + f time. We therefore conclude that by neglecting this small thermal contribution, workflows for this type of dyes can be sped up significantly.

Evaluation of the contribution to GSOP due to relaxation in the oxidized state, the main difference between the adiabatic and vertical approach, will follow in the next section.

### 4.2 Vertical approximations to GSOP

**Fig. 6:**
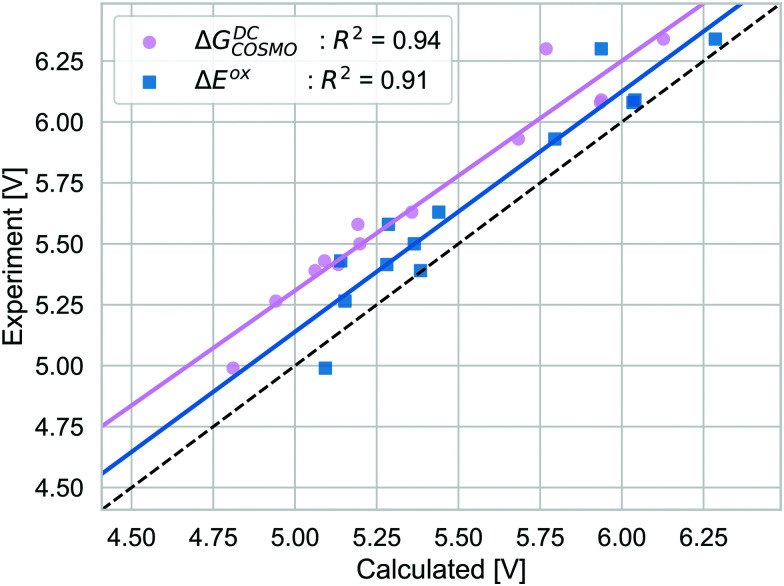
The GSOP computed as Gibbs free energy of the oxidation using the DC pathway (pink) and the vertical approach employing total electronic energy difference (blue) compared to the experimental oxidation potential (dashed line) *vs.* vacuum.

The alternative to the full adiabatic GSOP is to approximate it by a vertical oxidation potential. Apart from experimental considerations, such an approximation can always be justified if the effect of the geometry relaxation as well as other thermal contributions are negligible. The validity of this approximation can be assessed directly by comparing the Gibbs free energy for the oxidation reaction obtained with DC, Δ*G*^DC^_COSMO_ to the electronic energy difference Δ*E*^ox^: the calculations are performed in the same manner and we can focus on the physical approximations. The comparison is shown in Fig. 6.

Δ*E*^ox^ does include solvation effects (relaxation due to solvation as well) but it neglects the thermal contribution and the relaxation from the neutral to the oxidized geometry. The Δ*E*_ox_ data are close to the absolute experimental values with the MAD being (0.05 (RMSD = 0.18) eV). For Δ*E*^ox^ with *R*^2^ = 0.91, the strength of the linear correlation is slightly weaker than for the adiabatic approach. Comparing the differences between the two approaches, as discussed above, the thermal contributions (ESI,† S5) are rather small and consistent and should not affect the correlation strongly. In contrast, the difference coming from the relaxation due to oxidation is less consistent. On average, the energy difference due to using the oxidized or neutral geometry, both optimized in solution (gas) is 0.12 eV (0.15 eV) (ESI,† S5). The lower correlation for Δ*E*_ox_ is caused by a few dyes that show a larger change in energy between two oxidation states, especially NDI-5555 and NDI-555 for which the contributions due to this relaxation are 0.36 eV and 0.25 eV, respectively (ESI,† S5). However, the effect of the geometry relaxation after electron removal appears to be crucial.

**Fig. 7:**
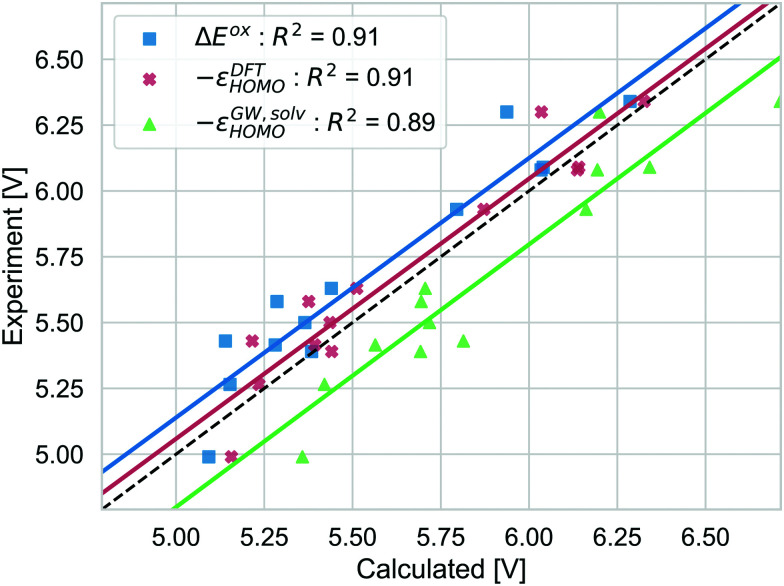
Computed vertical GSOPs with Δ*E*^ox^ (blue), −*ε*^DFT^_HOMO_ (red) and −*ε*^*GW*,solv^_HOMO_(green) compared to the experimental oxidation potential (dashed line) *vs.* vacuum.

Different strategies to calculate the vertical oxidation potential of the solvated dye are shown in Fig. 7. Δ*E*^ox^ (blue) and −*ε*^DFT^_HOMO_ give very similar results and also −*ε*^*GW*,solv^_HOMO_ gives a similar squared correlation coefficient. Compared to the other approaches, −*ε*^*GW*,solv^_HOMO_ overestimates the GSOP considerably, while Δ*E*^DFT^_ox_ (blue) and −*ε*^DFT^_HOMO_ underestimate it. This depends of course on the choice of the formalism to relate the reference electrode scale to the absolute scale. However, it is known that for the gas phase, partially self-consistent *GW* as well as *G*_0_*W*_0_ based on a functional with a high percentage of Hartree–Fock exchange often leads to overestimated IPs for organic molecules compared to experiment.^99^ On the other hand, using −*ε*^DFT^_HOMO_ from B3LYP, due to lower percentage of Hartree–Fock exchange should rather lead to underestimation of the oxidation potential.

The correlation with experiment becomes considerably better when the missing thermodynamic contributions are considered. Adding the energy lowering due to geometry relaxation to −*ε*^*GW*,solv^_HOMO_ significantly increases correlation with experiment, from *R*^2^ = 0.89 to *R*^2^ = 0.93 and for −*ε*^DFT^_HOMO_ and Δ*E*^ox^, the squared correlation coefficients increase from *R*^2^ = 0.91 to *R*^2^ = 0.96. These numbers should always be interpreted with the error bars in the experimental data in mind: differences in *R*^2^ of 0.01 (as for the difference between −*ε*^DFT^_HOMO_ and −*ε*^*GW*,solv^_HOMO_) are negligible, but the effect of structural relaxation is significant.

In summary, the data presented in this section suggests that the vertical approximation to GSOP is not reliable. It is irrelevant what particular method is used to calculate the vertical GSOP of the solvated dye. Reliable correlation with experiment is only achieved when the relaxation of the geometry after oxidation is accounted for. This is due to the fact that this contribution can be quite large for certain systems, but almost negligible for others, as illustrated for the example of NDI-5555. However, investigating the effect of thermal contributions, we found that they are very small and that their influence is negligible. In practice, this is very important since the frequency calculations required to calculate these contributions are computationally very demanding.

### 4.3 Timings

Since we want to find a method which is suitable for large-scale screening for potentially thousands of compounds, the CPU times of the different approaches are an important consideration. We use the unsubstituted PDI with 40 atoms as a representative example. Some of the systems considered here are about twice as large, but our conclusions do not change for these systems. All calculations presented in this subsection have been performed on a single 2.2 GHz intel Xeon (E5-2650 v4) node (broadwell architecture) with 24 cores and 128 GB RAM. Timings are shown in Table 1.

As already alluded to, Δ*G*^DC^_COSMO_ and Δ*G*^TC^_COSMO_ are by far the computational most demanding strategies, which is due to the expensive frequency calculations. Despite their good accuracy, they are clearly not suitable for large-scale compound screening. Calculation of the *G*_0_*W*_0_ QP energies is much cheaper but still more demanding than all other DFT-based strategies without frequency calculations. However, most of the time is spent on the QZ6P calculation. As explained in appendix 7, the *GW* calculations can also be performed with a TZ3P calculation only, without impairing the accuracy significantly. With the TZ3P calculation only, −*ε*^*GW*^_HOMO_ becomes competitive with Δ*E*^DFT^_ox_ and Δ*G*^TC^_COSMO-RS_ and all of these strategies could be considered for large scale-screening as well. Finally, Δ*G*^screening^_COSMO-RS_ is the computationally cheapest approach which is due to the fact that the geometry optimizations are performed on the GFN-xTB level. Since it provides an accuracy comparable to the involved Δ*G*^DC^_COSMO_ and Δ*G*^TC^_COSMO_ strategies, it is our method of choice for the screening of the dyes, described in the next subsection (Table 2).

**Table 2:** Statistical analysis^a^ of the considered strategies compared with cyclic voltammetry measurements in dichloromethane

Approach	MD	MAD	RMSD	*R* ^2^
Δ*G*^DC^_COSMO_	−0.28	0.28	0.30	0.94
Δ*G*^TC^_COSMO_	−0.45	0.45	0.46	0.95
Δ*G*^TC^_COSMO-RS_	−0.34	0.34	0.36	0.96
Δ*E*^ox^	−0.13	0.15	0.18	0.91
−*ε*^DFT^_HOMO_	−0.05	0.1	0.13	0.91
−*ε*^*GW*,solv^_HOMO_	0.20	0.22	0.24	0.89
−*ε*^*GW*,solv,geo^_HOMO_	0.03	0.08	0.12	0.93
Δ*G*^screening^_COSMO-RS_	−0.34	0.34	0.37	0.96

a MD stands for the mean deviation; MAD stands for the mean absolute deviation, RMSD stands for the root mean squared deviation; *R*^2^ is squared correlation.

### 4.4 Screening

**Fig. 8:**
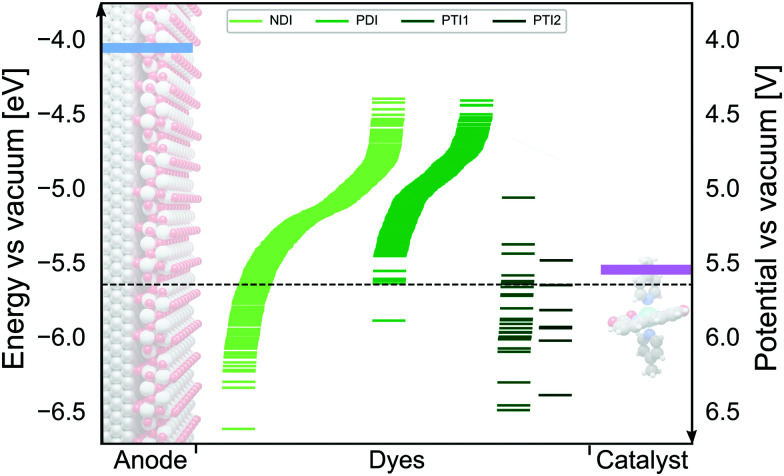
The alignment between oxidation energy levels (right axis) and potentials (left axis)of the DS-PECs components TiO_2_ CB edge (blue) and Ru-based^100^ WOC LOC (purple) with promising derivatives of four core molecules: NDI, PDI, PTI1 and PTI2 (green lines). The area under the dashed black line represents the desired GSOP values to match the highest oxidation potential of the chosen WOC.

We have employed the Δ*G*^screening^_COSMO-RS_ method to calculate the GSOP potentials of large number of dyes. We have applied it to set of dyes that we determined previously^17^ to have desirable optical properties. Among almost 2500 NDIs, PDIs, PTI1s and PTI2s derivatives around 1400 dyes fulfilled the criteria to be used as a photosensitizers in DS-PECs. All these dyes have an intense, lowest transition in the desired energy range, between the 1.35 eV which is the minimal thermodynamic requirement for water oxidation and 3.20 eV which is roughly the boundary between the visible and the UV energy range. During the water oxidation cycle the WOC goes through different oxidation states. For the GSOP of the dye the most important criteria is that it should lie higher than the WOC's highest oxidation potential (Fig. 1) to assure a favourable potential gradient for electron transfer. We thereby assume 0.1 V potential difference to be sufficient. As a trial WOC we will take the Ru-based catalyst designed by Duan *et al.*^100^ for which the most demanding catalytic step occurs at the potential 1.27 V *vs.* NHE (5.55 V *vs.* vacuum). Therefore, the lower limit for the dye's oxidation potential is at 5.65 V *vs.* vacuum (dashed black line in Fig. 8).

To better understand the effect of the root structure and the substituents, we looked at the range of GSOPs for the screened dye classes and at substituents that have the most extreme effect on the GSOP.

The unsubstituted NDI has a GSOP of 7.16 V *vs.* vacuum, which is lowered by substitution to a range of 6.61 V (NDI-4) to 4.40 V (NDI-5556) *vs.* vacuum. For the PDI, the GSOP without substitution is calculated to be 5.89 V *vs.* vacuum, while for the derivatives the GSOP range is from 5.64 V (PDI-4) to 4.41 V (PDI-6556) *vs.* vacuum. In both cases the considered substitutions are therefore lowering the GSOPs. This is not the case for the PTI1 and PTI2 core structures, where the calculated GSOPs are 6.31 V and 6.39 V respectively. For the PTI1 core the mono-substituted PTI1-4 and PTI1-1 have GSOP values of 6.49 V and 6.46 V *vs.* vacuum respectively, which are higher than that of the root structures. All other substituents are decreasing the GSOP value compared to the root structure, PTI1, with as lowest the PTI1-76 with a GSOP value of 5.07 V *vs.* vacuum. For the six PTI2 derivatives the lowest value is reached for PTI2-6, 5.49 V *vs.* vacuum, while the highest value is computed for monosubstituted PTI2-4, 6.03 V *vs.* vacuum.

For each of the root structures the ethyl alkoxide (4) substituent gives rise to the smallest GSOP shift and consequently the highest GSOP. For the NDI, PDI and PTI2 cores it decreases the value of GSOP the least compared to other substituents, while for PTI1 it increases the value of GSOP compared to unsubstituted PTI1. The substituents that appear to decrease the GSOP value the most are derived from ethanamine (5), pyrrole (6) and 4-ethynyl-*N*,*N*-dimethylaniline (7).

In Fig. 8 we show the calculated GSOP of 1340 dyes. The molecules under the dashed line fit the criterion that the GSOP is higher than the highest oxidation potential of the water oxidation catalyst, 5.65 V *vs.* vacuum (ESI,† S6). Out of the 118 dyes that fulfill the GSOP criterion 90 dyes are NDI derivatives, 21 PTI1 derivatives, 6 PTI2 derivatives and the final one is the PDI root structure. In contrast to this root structure, none of almost 500 PDI derivatives can fit this criterion. Of the suitable dyes the most common substituents in descending order are 4, 2 and 1. The substituents 5 and 6 appear less, while substituents 3 and 7 do not appear at all. These two substituents, derived from bithiophene and 4-ethynyl-*N*,*N*-dimethylaniline, are absent in the list of suitable dyes are decreasing the value of GSOP beyond the GSOP limit and thus prohibit the electron transfer from the WOC.

Considering only the catalyst criterion leaves a large number of candidate dyes, but one should keep in mind that the dye needs to also be able to inject an electron into the TiO_2_, which means that a promising dye should have an ESOP lower than the TiO_2_ conduction band. The leads to an additional criterion that can be used to further narrow down the set of potentially interesting dyes.

**Fig. 9:**
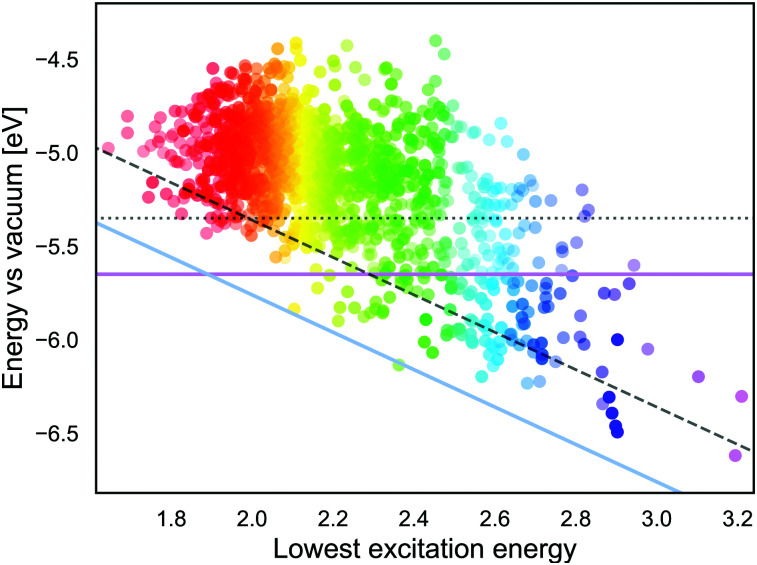
The lowest excitation energy and energy levels corresponding to GSOP potentials for the 1340 dyes. The color of the dot corresponds to its absorption energy and its opacity corresponds to oscillator strength normalised to 0 to 1 scale. The horizontal purple line represents the experimental GSOP limit and dotted line (parallel to purple line) includes computational deviations. The blue line represents the experimental ESOP limit (the sum of GSOP and *λ*_max_ needs to exceed the TiO_2_ CB). The dashed line (parallel to blue line) includes computational deviation. The optimal set of dyes are in between the purple and dashed line.

If we approximate the ESOP as GSOP + *λ*_max_, the energy corresponding to the ESOP needs to be higher than the energy corresponding to the TiO_2_ CB. As mentioned in the Introduction, for an optimal rate of the electron transfer ESOP should be approximately 0.3 V higher than the CB edge. Fig. 9 shows energy levels corresponding to GSOP and the lowest excitation energies (from ref. 17) for the dyes that fulfil absorption criteria. The purple line is the upper limit for energy that corresponds to the dyes GSOP. The dyes above the blue line satisfy the criteria that the sum GSOP + *λ*_max_ is higher than the energy corresponding to ESOP limit, 4.36 V *vs.* vacuum (TiO_2_ CB is 4.00 V *vs.* vacuum). A trivial way to include the expected deviations of the computed values compared to the experimental ones, is to shift the criteria for the value of MD. The dotted line, (parallel to the purple horizontal line) is the limit for GSOP including the underestimations of experimental oxidation potentials, the MD of about 0.3 V. The dashed line (parallel to the blue diagonal line) takes into account also the overestimation of the experimental absorption properties, which has a MD of about 0.4 V. The dyes in between the purple line and dashed line therefore represent the set of dyes that are suitable to this particular example system, taking into account errors in the computations. This reduces the set of suitable dyes to 66 dyes. From this set we show the dyes that have the highest oscillator strength in Table 3. The full list of these dyes is also indicated in the ESI,† S6.

**Table 3:** GOSP [*V vs.* vacuum], lowest most intense excitation *λ*_max_ [eV] and oscillation strength (osc.str) values for 10 suitable dyes with the highest oscillation strengths^17^

Name	GSOP	*λ*_max_	osc.str.
PTI1-5	6.00	2.90	0.66
PTI1-144	5.88	2.67	0.58
PTI1-444	5.81	2.67	0.56
PTI1-544	5.91	2.67	0.55
PTI2-4	6.03	2.70	0.55
PTI2-44	5.82	2.64	0.54
PTI1-54	6.02	2.72	0.53
NDI-125	5.78	2.57	0.45
NDI-225	5.84	2.49	0.44
PTI1-15	5.73	2.49	0.44

## 5 Conclusions

We have computed the solution-phase Gibbs free energy, with adiabatic and vertical approaches, employing different methods. First we have built the experimental data set from the CV measurements for the derivatives of the NDI and PDI dyes to validate the computational results. Calculations of solution-phase Gibbs free energy are done at the DFT level using different CSM pathways, the DC and TC pathways, and using different CSM models – COSMO and COSMO-RS. We find that, to calculate the ground state oxidation potential for these dyes, both pathways using the COSMO model perform well. The TC path shows a slightly higher value of squared correlation with the experiment, but a higher MAD value as well. The TC pathway with the COSMO-RS model has the highest correlation with the experiment with a MAD lower than the TC pathway with COSMO. Comparison to different vertical approximation, Δ*E*^ox^ and one electron energy strategies, the −*ε*^DFT^_HOMO_ and −*ε*^*GW*^_HOMO_, shows the importance of taking into account geometry relaxation after oxidation, as well as the inclusion of electronic solvent effects. However, other thermal effects do not play an important role when it comes to this set of molecules. Therefore, the adiabatic approach appears more suitable for screening purposes, but one should be aware that this procedure might be unfavourable for the molecules with large number of conformational isomers that exist in a small energy range and that are highly affected by solvent, such as PDI-0000. Replacing DFT geometry optimization by SQM optimization has not significantly affected the correlation with experiment for the adiabatic approach combined with the COSMO-RS model, while it reduced the cost of the strategy by a factor of eight. Therefore, the Δ*G*^screening^_COSMO-RS_ is found to be a suitable strategy for screening on a desired GSOP range for derivatives of the NDI and PDI dyes.

The dyes that have been proposed as suitable for panchromatic sensitization of the photoelectrode in DS-PECs have been further characterised for their redox properties, in particular GSOP. Using the Δ*G*^screening^_COSMO-RS_ strategy, the GSOP is evaluated for 1340 dyes. For the system of choice, which is a TiO_2_ based photoelectrode and Ru-based catalyst^100^ as a WOC, there are 118 dyes that fulfil the given criteria.

## Conflicts of interest

There are no conflicts to declare.

## Supplementary Material

CP-024-D1CP04218A-s001

## Figures and Tables

**Fig. 10:**
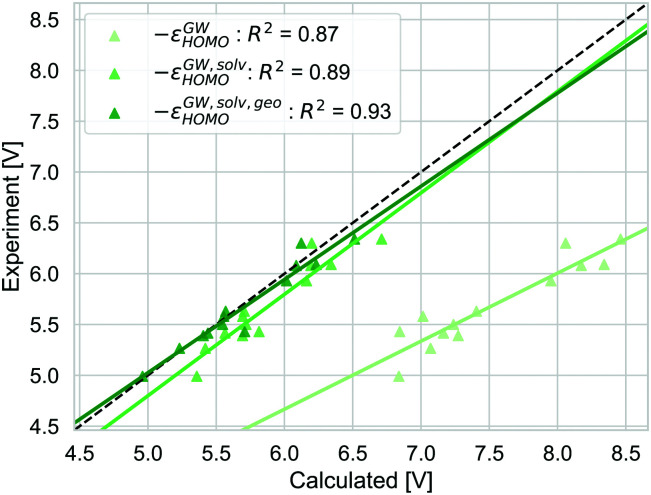
The GSOP calculated with the GW approaches successively including more physical effects (solvation effects and geometry relaxation due to oxidation): −*ε*^*GW*^_HOMO_, −*ε*^*GW*,solv^_HOMO_ and −*ε*^*GW*,solv,geo^_HOMO_ (lightest to darkest shade of green) compared to the experimental oxidation potential (dashed line) *vs.* vacuum.

## Appendices

### A Effect of solvent and structural relaxation on *GW*

The output of the *GW* calculations are QP energies. They correspond to the vertical electron addition and removal energies in vacuum and can not be compared to the GSOP directly. This is shown here for *G*_0_*W*_0_@PBE0(HF = 0.4) in Fig. 10, the same is observed for all other tested *GW* approaches. Identifying −*ε*^*GW*^_HOMO_ systematically overestimates the GSOP by several eV on average. Also the squared correlation is with only *R*^2^= 0.87 worse than for Δ*G*. Accounting for solvent effects removes most of the overestimation and improves *R*^2^ to 0.89. Taking into account the structural relaxation after electron removal improves the correlation to *R*^2^ = 0.93, which is now competitive with Δ*G*. Note, that this does not include other thermal contributions. As discussed above, they are very small and can be neglected. Our results are consistent with earlier work by Umari *et al.*^37^ who compared *G*_0_*W*_0_@BLYP vertical oxidation potentials to experimental data from differential pulse voltammetry in acetonitrile.^101^ They observed that their *GW* approach overestimated the experimental values by nearly one eV on average, even though it is known, that *G*_0_*W*_0_ based on a GGA starting point systematically underestimates the gas phase oxidation potential of organic molecules.^99^

### B Comparison and validation of *GW* methods

Taking into account these considerations, we can compare the different *GW* methods to experiment. We tested all approaches using the TZ2P and TZ3P basis sets and calculated the *R*^2^ values with respect to experiment. The result of our comparison is shown in Table 4. The correlation of *G*_0_*W*_0_ was found to be more or less independent of the starting point. Eigenvalues-only self-consistency does not improve the results compared to *G*_0_*W*_0_ for TZ3P and worsens them for TZ2P. The same is observed for qs*GW*. qs*GW*_0_ gives much worse correlation coefficient on the TZ3P level (*R*^2^ = 0.85) than the other methods. For this reason, using one of the more expensive partially self-consistent approaches is not justified and we used the *G*_0_*W*_0_PBE0(HF = 0.4) method for comparison to the other computational methods in the main body of the text. Our conclusion that self-consistency is not needed here is of course not valid in general and in other cases it might be possible that updates in the wave-function are needed. Reordering of orbitals during the *GW* calculation can be an indicator for this and in such cases it might be necessary to use some kind of self-consistency in *GW*.^36^

**Table 4:** The squared correlation coefficients *R*^2^ compared to experiment for different *GW* approaches for different basis sets. Solvent corrections and the effects of geometry relaxation are included

Method	TZ2P	TZ3P
*G*_0_ *W*_0_@PBE0(HF = 0.4)	0.91	0.92
*G*_0_ *W*_0_@LRC *ω* PBEH	0.92	0.91
ev*GW*@PBE0(HF = 0.4)	0.87	0.92
ev*GW*@*LRCω*PBEH	0.89	0.90
qs*GW*_0_@PBE	0.88	0.85
qs*GW*	0.86	0.91

For optimal agreement with experiment, the basis set error needs to be removed from the QP energies as well, using eqn (10). However, for the molecules considered here, basis set limit extrapolation increases the correlation of *G*_0_*W*_0_PBE0(HF = 0.4) with experiment only marginally from *R*^2^ = 0.92 to *R*^2^ = 0.93. This is due to the fact that CBS limit extrapolation results in a more or less constant shift of the QP energies, which then leaves the correlation coefficient mainly unaffected.
