# Correction: Automated assessment of redox potentials for dyes in dye-sensitized photoelectrochemical cells†

**DOI:** 10.1039/d3cp90114a

**Published:** 2023-06-02

**Authors:** Jelena Belić, Arno Förster, Jan Paul Menzel, Francesco Buda, Lucas Visscher

**Affiliations:** a Department of Chemistry and Pharmaceutical Sciences, Vrije Universiteit Amsterdam De Boelelaan 1083 1081 HV Amsterdam The Netherlands l.visscher@vu.nl; b Leiden Institute of Chemistry, Leiden University Einsteinweg 55, P.O. Box 9502 2300 RA Leiden The Netherlands

## Abstract

Correction for ‘Automated assessment of redox potentials for dyes in dye-sensitized photoelectrochemical cells’ by Jelena Belić *et al.*, *Phys. Chem. Chem. Phys.*, 2022, **24**, 197–210, https://doi.org/10.1039/D1CP04218A.

The authors have found an error in processing the components of the solvation energy in the published version of this manuscript. The Gibbs free energy of solvation was missing a contribution from the energy required to polarize the solute. While the equations are correct, the values attributed to them are not. This error led to a consistent shift by 0.1 eV on average, in the values for the reported solvation energies and Gibbs free energies calculated *via* the TC and GW approaches that include solvation effects. However, as the conclusions were based on the extent of the linear relationship between the experimental and theoretical values, this error did not affect the main conclusions. Tables and Figures that contain the error in the original publication and the Electronic Supplementary Information (ESI) are Tables 2, S2, S3, S4 and S5 and Fig. 5, 7 and 10. The changes in the Tables and Figures from the original publication have been summarised below with the corrections for the corresponding Tables and Figures. Please refer to the revised ESI (https://www.rsc.org/suppdata/d1/cp/d1cp04218a/d1cp04218a1.pdf) for the correction in the ESI tables.

**Table:** Statistical analysis^a^ of the considered strategies compared with cyclic voltammetry measurements in dichloromethane

Approach	MD	MAD	RMSD	*R* ^2^
Δ*G*^DC^_COSMO_	−0.28	0.28	0.30	0.94
Δ*G*^TC^_COSMO_	**−0.35**	**0.35**	**0.36**	**0.94**
Δ*G*^TC^_COSMO-RS_	−0.34	0.34	0.36	0.96
Δ*E*^ox^	−0.13	0.15	0.18	0.91
–*ε*^DFT^_HOMO_	−0.05	0.10	0.13	0.91
–*ε*^GW,solv^_HOMO_	**0.32**	**0.32**	**0.34**	**0.91**
−*ε*^GW,solv,geo^_HOMO_	**0.15**	**0.16**	**0.19**	**0.95**
Δ*G*^screening^_COSMO_	−0.34	0.34	0.37	0.96

a MD stands for the mean deviation; MAD stands for the mean absolute deviation, RMSD stands for the root mean squared deviation; *R*^2^ is squared correlation.

**Fig. 5:**
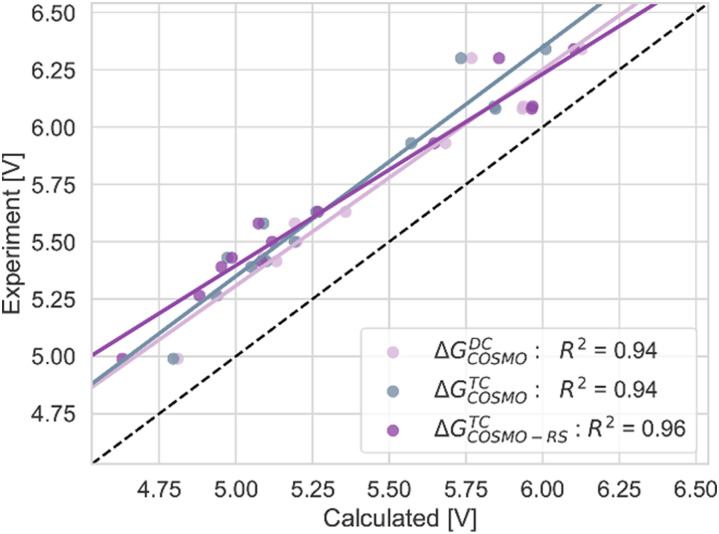
The correlation of adiabatic GSOP computed with Δ*G*^TC^_COSMO_ (pink), Δ*G*^DC^_COSMO_ (grey) and Δ*G*^TC^_COSMO-RS_ (purple) methods to the experimental oxidation potential *vs.* vacuum (dashed line).

**Fig. 7:**
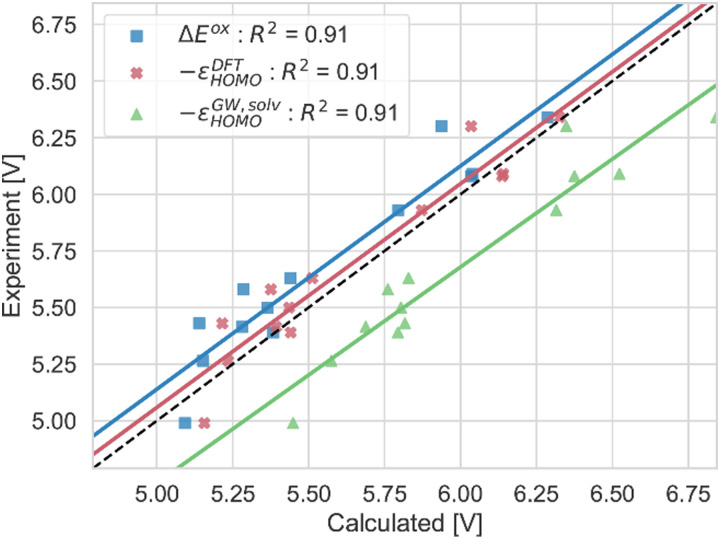
Computed vertical GSOPs with *AE*^ox^ (blue), –*ε*^DFT^_HOMO_ (red) and –*ε*^GW,solv^_HOMO_ (green) compared to the experimental oxidation potential (dashed line) *vs.* vacuum.

**Fig. 10:**
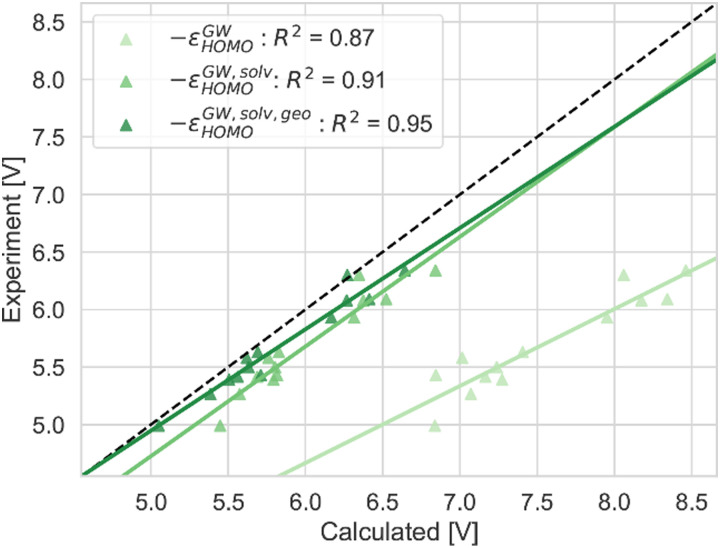
The GSOP calculated with the GW approaches successively including more physical effects (solvation effects and geometry relaxation due to oxidation): –*ε*^GW^_HOMO_, –*ε*^GW,solv^_HOMO_ and −*ε*^GW,solv,geo^_HOMO_ (lightest to darkest shade of green) compared to the experimental oxidation potential (dashed line) *vs.* vacuum.

In Table 2, the corrected values of MD, MAD, and RMSD (in bold) manifest a shift for the Gibbs free energy values by 0.1 eV on average. The correlation of *R*^2^ with the experiments for the Δ*G*^TC^_COSMO_ decreased by 0.01 while for –*ε*^GW,solv^_HOMO_ and −*ε*^GW,solv,geo^_HOMO_ it increased by 0.02. These slight changes affect the text referring to the values in the Tables and Figures. Particularly in the Conclusion, the correct analysis is: “We find that, to calculate the ground state oxidation potential for these dyes, both pathways using the COSMO model perform well. The TC and DC pathways show the same value of squared correlation with the experiment, where the TC path shows a higher MAD value”.

In Table S2, the corrected values for Gibbs free energies calculated *via* the TC approach are given.

In Table S3, corrected values for Gibbs free energies calculated GW approaches that include solvation effects are given.

In Table S4, for the case of the PDI-0000 molecule, the corrected values for Gibbs free energies calculated with the TC approach are given.

Table S5 shows the solvation contribution to the Gibbs free energies calculated with the TC and DC approach. All values are corrected, and the correction of the text that refers to this table is: “On average, the value of ΔΔ*G* is −1.50 eV, with a maximum value of −1.80 eV for NDI-58.”

In Fig. 5, 7, and 10, the *R*^2^ values are corrected according to the values in Table 2.

The Royal Society of Chemistry apologises for these errors and any consequent inconvenience to authors and readers.

## Supplementary Material

