# Supplementary material for: Automated assessment of redox potentials for dyes in dye-sensitized photoelectrochemical cells
Source: Phys Chem Chem Phys. 2021 Dec 1;24(1):197–210. doi: 10.1039/d1cp04218a (PMC8694061; doi:10.1039/d1cp04218a)
Supplement: CP-024-D1CP04218A-s001 [file CP-024-D1CP04218A-s001.pdf]

Electronic Supplementary Material (ESI) for Physical Chemistry Chemical Physics.
This journal is © the Owner Societies 2021

Supporting Information: Automated assessment of redox potentials for dyes in dye-sensitized photoelectrochemical cells

Jelena Belić,*,† Arno Förster,† Jan Paul Menzel,‡ Francesco Buda,‡ and Lucas Visscher*,†

†*Department of Chemistry and Pharmaceutical Sciences, Vrije Universiteit Amsterdam, De Boelelaan 1083, 1081 HV, Amsterdam, The Netherlands*

‡*Leiden Institute of Chemistry, Leiden University, Einsteinweg 55, P.O. Box 9502, 2300 RA, Leiden, The Netherlands*

E-mail: j.belic@vu.nl; l.visscher@vu.nl

Contents

S1 Experimental data set **S-3**

S2 R^2 sensitivity analysis **S-5**

S3 Calculation steps of the GSOP strategies and calculated GSOP values **S-6**

S4 PDI-0000 outlier analysis **S-8**

S5 Different contributions to the GOSP in the adiabatic approach (the Gibbs free energy of the oxidation reaction) **S-10**

S6 Suitable dyes for the trial system containing the Ru-based water oxidation catalyst designed by Duan et al.[S1] and TiO_2 anode. **S-14**

References **S-18**

S1 Experimental data set

The experimental data has been collected from different sources, presented in the Table S1. For computational convenience both core (numbered C atoms of the aromatic core) and imide substituents are slightly modified (Figure S1). All the imide substituents at NDI and PDI derivatives are replaced with a hydrogen atom. We compared experimentally obtained oxidation potentials of two NDIs substituted at the core with alkyl chains of different length. In a paper by Röger and Würthner, we note the difference between oxidation potential of two tetraalkylamino NDIs.[S2] The two NDI derivatives bearing the cyclic diaminoethyl and hexylamino had oxidation potentials of -0.01 V and 0.01 V, respectively. Therefore, the shortening of the alkyl chain resulted in a decrease of the oxidation potential of 0.02 eV. Thalacker, Röger and Würthner reported[S3] oxidation potential for di-substituted NDI core with octylamino groups of 0.60 V and di-substituted NDI core with one octylamino and one 4-tert-butylphenylamino group of 0.62 V. In this case the oxidation potential increased for 0.02 V with the introduction of an aryl group at one of the substitution spots.

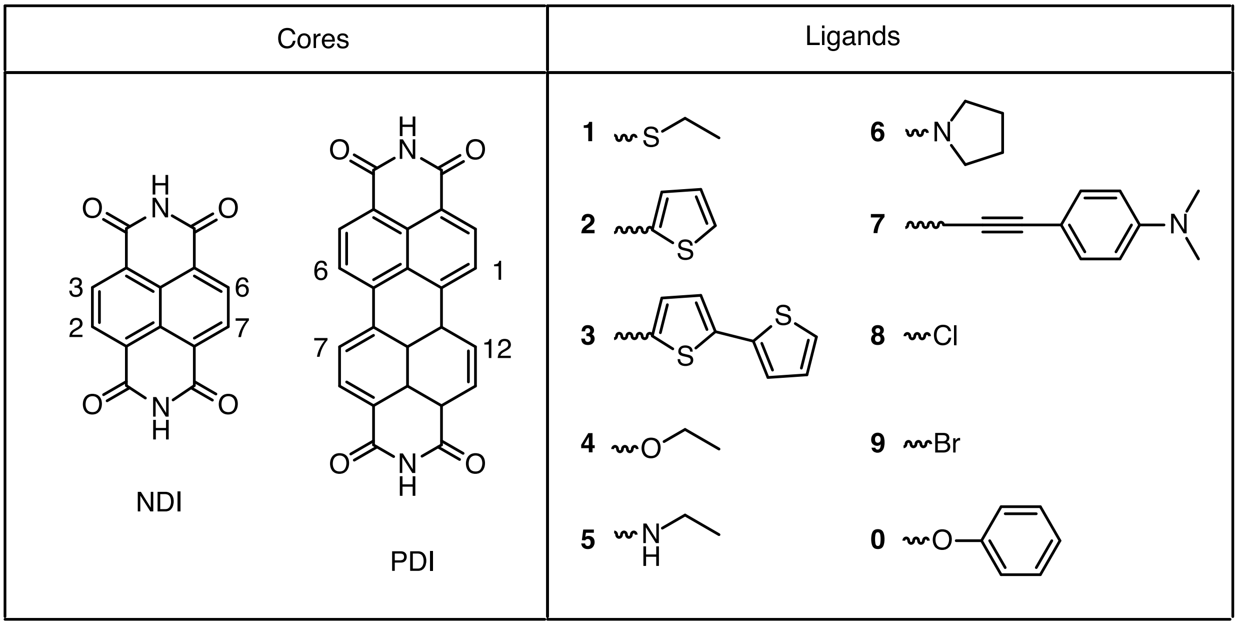

Figure S1: Structures of the unsubstituted cores that have been used for validation of the computational results and structures of all the ligands in the paper

The NDI core is substituted in the following order, first position 2, than 6, 3 and 7. If represented as a list, the first element of the list corresponds to substitution on position 2, the second element corresponds to substitution position 6, *etc.*; if we use numbers to represent substituents as in Figure S1, we can represent NDI derivatives in a short notation as NDI-11 for 2,6-di-alkylthio-substituted NDI. The PDI core is substituted in the analogous way at the positions 1, 7, 6 and 12. The PDI-55c and PDI-55t are conformational isomers, where c and t stand for *cis* (on the same side) and *trans* (on the other side), which means that PDI-55c is substituted at the positions 1 and 6 while PDI-55c at the positions 7 and 12. The experimental values came from different sources; we used raw experimental data obtained in very similar conditions. All values are obtained with cyclic voltammetry (CV) in dichloromethane (DCM) with a scan rate of 100 mVs^{-1} (except NDI-59, 20 mVs^{-1}) and with tetrabutylammonium hexafluorophosphate (NBu_4PF_6) as a supporting electrolyte. For NDI-77 and NDI-66 the oxidation potentials are determined against an SCE electrode and for other dyes against the ferrocene/ferricenium couple Fc/Fc^+ as internal standard (Ag/AgCl reference electrode).

Here we define absolute potential for used reference electrodes. The reported value for the Fc/Fc^+ potential is 0.46 V versus SCE in DCM solution (0.1 M NBu_4PF_6).[S10,S11] The standard value of SCE versus normal hydrogen electrode (NHE), which is by Bard[S12] considered the same as standard hydrogen electrode (SHE) is 0.24 V. Finally, we can relate the SHE reference electrode potential to the absolute scale. Sticking to the Fermi-Dirac statistics for the electron convention, calculated absolute potential for the SHE reference electrode is 4.28 V.[S13,S14] Therefore, knowing the relations between the electrodes, the absolute potential for the SCE electrode is 4.52 V and for Fc/Fc^+ 4.98 V.

Table S1: Experimentally measured half-wave potentials, $E_{1/2}$, for core-substituted NDI and PDI dyes. The potentials are obtained with cyclic voltammetry in dicloromethane, with NBu_4PF_6 as a supporting electrolyte at scan rate of 100 mVs^{-1} (except NDI-59, 20 mVs^{-1}) referenced against Fc/Fc^+ or SCE. The reported values are converted to absolute scale[a]

Name	$E_{1/2}$ [V] vs (Reference Electrode)	$E^{abs}_{1/2}$ [V]	Ref.
NDI-44	1.36 (Fc/Fc^+)	6.34	S4
NDI-54	0.95 (Fc/Fc^+)	5.93	S5
NDI-55	0.65 (Fc/Fc^+)	5.63	S2
NDI-555	0.41 (Fc/Fc^+)	5.39	S2
NDI-5555	0.01 (Fc/Fc^+)	4.99	S2
NDI-58	1.11 (Fc/Fc^+)	6.09	S6
NDI-59	1.10 (Fc/Fc^+)	6.08	S6
NDI-66	0.98 (SCE)	5.50	S7
NDI-77	0.91 (SCE)	5.43	S7
PDI	1.32 (Fc/Fc^+)	6.30	S8
PDI-4444	0.60 (Fc/Fc^+)	5.58	S8
PDI-55c	0.44 (Fc/Fc^+)	5.42	S9
PDI-55t	0.29 (Fc/Fc^+)	5.27	S9
PDI-0000	0.93 (Fc/Fc^+)	5.91	S8

[a] $E^{abs}_{1/2} = 4.98$ V (Fc/Fc$^+$) + $E_{1/2}$; $E^{abs}_{1/2} = 4.52$ V(SCE) + $E_{1/2}$.

S2 R^2 sensitivity analysis

We want to check the sensitivity of the R^2 to the error of ± 0.05 eV to the experimental values. We do that by adding random values from the interval (-0.05, 0.05) eV to the experimental values to randomize the data set. We then iteratively calculate the value of R^2 between the calculated values and randomised data and calculate the MAD for the R^2. We repeat this process increasing the number of iterations to avoid initialisation noise. The MAD converges to the value of 0.01.

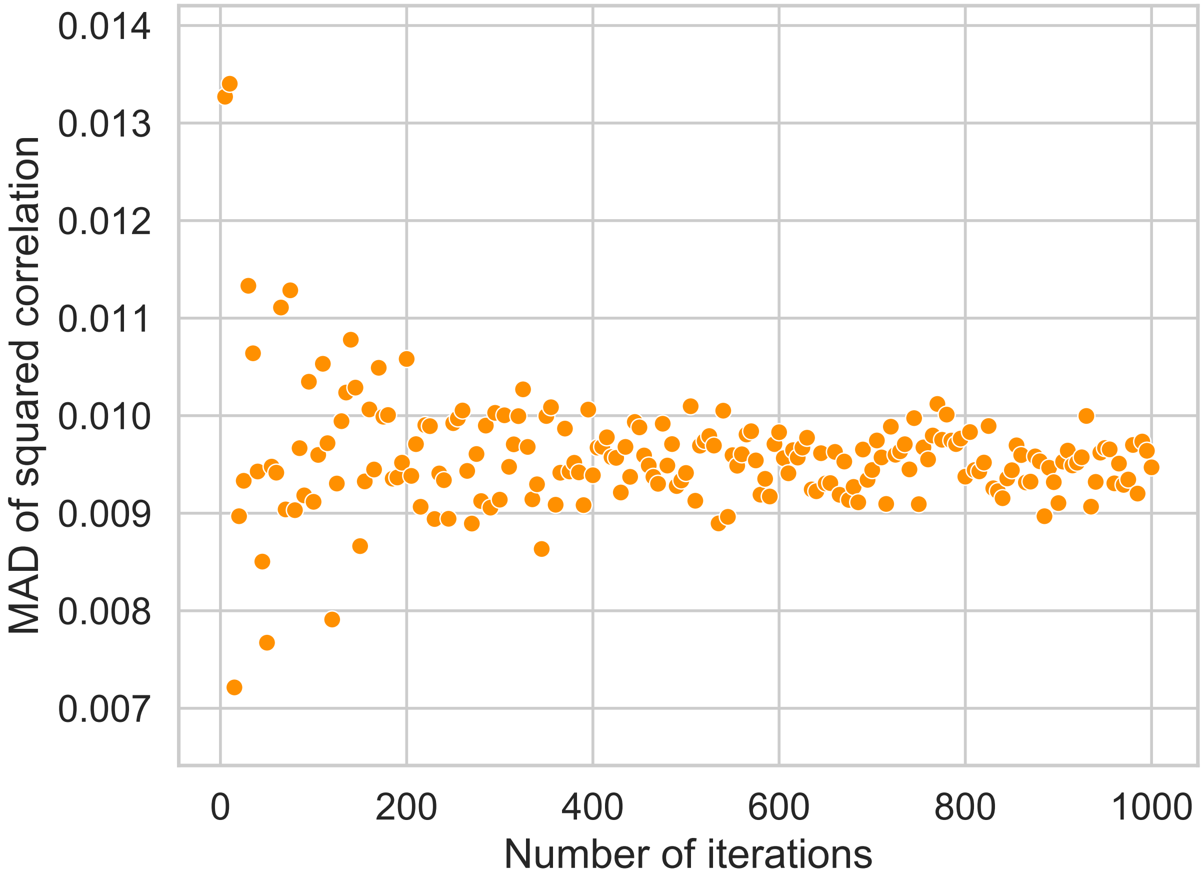

Figure S2: The mean absolute deviation for R^2 due to 0.05 eV error for increasing number of iterations.

S3 Calculation steps of the GSOP strategies and calculated GSOP values

Direct approach with COSMO includes two steps:

1. Solution-phase geometry optimization with frequencies for the neutral, $G^0_{sol}(g^0_{sol})$ and

2. Solution-phase geometry optimization with frequencies for the oxidized, $G^+_{sol}(g^+_{sol})$ molecule.

From these calculations we can directly obtain the Gibbs free energies, as in Equation (S1).

$$\Delta G^{DC}_{COSMO} = G^+_{sol}(g^+_{sol}) + G_{gas}(e^-) - G^0_{sol}(g^0_{sol}) \tag{S1}$$

$G^{+}_{sol}(g^{+}_{sol})$ and $G^{0}_{sol}(g^{0}_{sol})$ are solution-phase Gibbs free energies of the oxidized and neutral molecular species, respectively.

Thermodynamic cycle approach with COSMO (or COSMO-RS) requires nine calculations shown below to obtain components of equation (S2), where i denotes neutral (0) and oxidized (+) molecule. The ΔG^{TC}_{COSMO} is calculated as in equation (S3):

$$G^{i}_{sol}(g^{i}_{gas}) = G^{i}_{gas}(g^{i}_{gas}) + \Delta G^{i}_{solv}(g^{i}_{gas}) + [E_{gas}(g^{i}_{sol}) - E_{gas}(g^{i}_{gas})] \quad \text{(S2)}$$

$$\Delta G^{TC}_{COSMO} = G^{+}_{sol}(g^{+}_{gas}) + G_{gas}(e^{-}) - G^{0}_{sol}(g^{0}_{gas}) \quad \text{(S3)}$$

For the neutral molecule:

1. Gas-phase geometry optimization with frequencies, $G^{0}_{gas}(g^{0}_{gas})$ and $E_{gas}(g^{0}_{gas})$
2. Single point on gas-phase geometry in solution, $\Delta G^{0}_{solv}(g^{0}_{gas})$
3. Solution-phase geometry optimization, geometry (g^{0}_{sol})
4. Single point on solution-phase geometry in gas, $E_{gas}(g^{0}_{sol})$

For oxidized molecule:

1. Gas-phase geometry optimization with frequencies with charge $G^{+}_{gas}(g^{+}_{gas})$
2. Single point on gas-phase geometry in solution with charge, $\Delta G^{+}_{solv}(g^{+}_{gas})$
3. Single point on gas-phase geometry in gas, $E_{gas}(g^{+}_{gas})$
4. Solution-phase geometry optimization with charge, geometry (g^{+}_{sol})
5. Single point on solution-phase geometry in gas, $E_{gas}(g^{+}_{sol})$

Thermodynamic cycle with COSMO-RS used for screening includes six steps in total. For this approach three calculations are needed to calculate the solution-phase Gibbs free energy $G^{i}_{sol,CRS}(g^{i}_{gas})$ for $i = 0, +$ state (Equation (S4)). The Gibbs free energy of oxidation is given in Equation (S5)

$$G^{i}_{sol,CRS}(g^{i}_{gas}) = E^{i}_{sol}(g^{i}_{gas}) + \Delta G^{i}_{CRS,solv}(g^{i}_{gas}) \quad \text{(S4)}$$

$$\Delta G^{screening}_{COSMO-RS} = G^{+}_{sol,CRS}(g^{+}_{gas}) + G_{gas}(e^{-}) - G^{0}_{sol,CRS}(g^{0}_{gas}) \quad \text{(S5)}$$

1. Geometry optimisation in gas-phase with semiempirical quantum mechanical (SQM) techniques, g^{i}_{gas}

2. Single point on gas-phase geometry in solution (COSMO) on DFT level, $E^{i}_{sol}(g^{i}_{gas})$

3. COSMO-RS calculation $G^{i}_{sol,CRS}(g^{i}_{gas})$

Table S2: The GSOP evaluated with adiabatic approach with different strategies. The values are calculated using the Equation (S1), (S3) and (S5). All values are in eV.

Name	EXP	ΔG^{DC}_{COSMO}	ΔG^{TC}_{COSMO}	$\Delta G^{TC}_{COSMO-RS}$	$\Delta G^{screening}_{COSMO-RS}$
NDI-44	6.34	6.13	5.89	6.10	6.14
NDI-54	5.93	5.68	5.45	5.65	5.66
NDI-55	5.63	5.36	5.16	5.27	5.27
NDI-555	5.39	5.06	4.96	4.95	4.92
NDI-5555	4.99	4.81	4.73	4.63	4.61
NDI-58	6.09	5.94	5.68	5.97	5.97
NDI-59	6.08	5.93	5.68	5.97	5.97
NDI-66	5.50	5.20	5.12	5.12	5.14
NDI-77	5.43	5.09	4.97	4.99	4.95
PDI	6.30	5.77	5.60	5.86	5.86
PDI-55c	5.42	5.13	4.98	5.08	5.06
PDI-55t	5.27	4.94	4.81	4.88	4.85
PDI-4444	5.58	5.19	5.03	5.07	5.07
PDI-0000	5.91	5.20	5.08	5.26	5.48

S4 PDI-0000 outlier analysis

Following the automated procedure for ΔG^{DC}_{COSMO} and ΔG^{TC}_{COSMO} strategy to calculate adiabatic GSOP, molecule PDI-0000 appears as an outlier. We analyse the effective contributions to the GSOP for the two strategies such as thermal contributions, geometry relaxation due to solvation and due to oxidation. These effects are not very different compared to other molecules, therefore do not have large effect on the value of the GSOP. There is a slight differ-

Table S3: The GSOP evaluated with vertical approach with different strategies. All values are in eV.

Name	EXP	$-\epsilon_{HOMO}^{GW}$	$-\epsilon_{HOMO}^{GW,solv}$	$-\epsilon_{HOMO}^{GW,solv,geo}$	ΔE^{ox}	$-\epsilon_{HOMO}^{DFT}$
NDI-44	6.34	8.46	6.71	6.51	6.29	6.33
NDI-54	5.93	7.95	6.16	6.02	5.80	5.87
NDI-55	5.63	7.41	5.71	5.57	5.44	5.51
NDI-555	5.39	7.27	5.69	5.41	5.38	5.44
NDI-5555	4.99	6.84	5.36	4.96	5.09	5.16
NDI-58	6.09	8.34	6.34	6.23	6.04	6.14
NDI-59	6.08	8.17	6.19	6.09	6.03	6.14
NDI-66	5.50	7.24	5.72	5.55	5.37	5.44
NDI-77	5.43	6.84	5.81	5.71	5.14	5.22
PDI	6.30	8.06	6.20	6.12	5.94	6.04
PDI-55c	5.42	7.16	5.56	5.44	5.28	5.39
PDI-55t	5.27	7.07	5.42	5.23	5.15	5.24
PDI-4444	5.58	7.01	5.69	5.56	5.29	5.38
PDI-0000	5.91	7.24	5.92	5.83	5.55	5.64

ence compared to the $\Delta G_{COSMO-RS}^{TC}$ due to the the change in solvation model, COSMO-RS instead of COSMO. We notice a somewhat larger difference in the $G_{COSMO-RS}^{screening}$ model where, compared to $\Delta G_{COSMO-RS}^{TC}$, geometry optimization is performed with GFN1-xTB instead of DFT, Table S4. Würthner[S15] mentions that the tetraphenoxy-substituted PDI preferred conformation in gas is unclear as many conformational isomers exist in a small energy range, therefore the conformation in solution will highly depend on solvent effects. For similar molecules it has been shown[S8] that the molecule is twisted with the torsion angle around 25 degrees between two naphtalene planes which are forming the PDI core. The procedure we applied, does not employ conformational search and we assume that the molecule ended up in one of the local minima. In addition, we notice that the bonding energy in solvent calculated with COSMO (DC and TC strategies) for the oxidized molecule on oxidized geometry is larger than for the oxidized molecule on neutral geometry by 0.23 eV, while the energy difference induced only by geometry change due to oxidation is 0.05 eV as shown in Table S8, which describes the sensitivity of the molecule's electronic energy to the solvent

model. Therefore, we conclude that the discrepancy from the experimental value originates in the molecule's sensitivity to the environment in combination with the conformational uncertainties.

Table S4: The GSOP evaluated with different strategies for PDI-0000 molecule and the absolute deviation (AD) of PDI-0000 compared to the mean absolute deviation (MAD) for the rest of the data set for given strategy. All values are in V.

	ΔG^{DC}_{COSMO}	ΔG^{TC}_{COSMO}	$\Delta G^{TC}_{COSMO-RS}$	$\Delta G^{screening}_{COSMO-RS}$
GSOP	5.20	5.08	5.26	5.48
AD	0.67	0.80	0.65	0.43
MAD	0.28	0.45	0.34	0.34

S5 Different contributions to the GOSP in the adiabatic approach (the Gibbs free energy of the oxidation reaction)

Table S5: The solvation contribution to the GSOP for the two methods, DC and TC.

Name	$\Delta\Delta G_{solv}^{DC}$ [eV][a]	$\Delta\Delta G_{solv}^{TC}$ [eV][b]
NDI-44	-1.75	-1.73
NDI-54	-1.79	-1.76
NDI-55	-1.70	-1.68
NDI-555	-1.58	-1.56
NDI-5555	-1.48	-1.46
NDI-58	-2.00	-1.97
NDI-59	-1.98	-1.95
NDI-66	-1.52	-1.51
NDI-77	-1.03	-1.02
PDI	-1.86	-1.84
PDI-4444	-1.32	-1.31
PDI-55c	-1.60	-1.59
PDI-55t	-1.65	-1.62
PDI-0000	-1.30	-1.28

[a] $\Delta\Delta G_{solv}^{DC} = \Delta G_{solv}^{+}(g_{sol}^{+}) - \Delta G_{solv}^{0}(g_{sol}^{0})$
[b] $\Delta\Delta G_{solv}^{TC} = \Delta G_{solv}^{+}(g_{gas}^{+}) - \Delta G_{solv}^{0}(g_{gas}^{0})$

Table S6: Thermal contributions to the GSOP, for the direct method (frequencies performed in solution phase) and for TC method (frequencies performed in the gas phase), at the $T = 298.15K$. The average contribution of the absolute values of ΔG^{DC}_{therm} and ΔG^{TC}_{therm} is 0.04 and 0.03 respectively.

Name	ΔG^{DC}_{therm} [eV][a]	ΔG^{TC}_{therm} [eV][b]
NDI-44	0.05	-0.02
NDI-54	0.05	-0.02
NDI-55	0.07	0.01
NDI-555	0.02	0.02
NDI-5555	0.09	0.06
NDI-58	0.03	-0.03
NDI-59	0.03	-0.02
NDI-66	0.01	0.02
NDI-77	0.04	-0.08
PDI	-0.07	-0.07
PDI-4444	0.05	-0.02
PDI-55c	-0.01	-0.02
PDI-55t	-0.002	-0.004
PDI-0000	-0.08	-0.05

[a] $\Delta G^{DC}_{therm}(T) = G^{+}_{therm}(g^{+}_{sol}, T) - G^{0}_{therm}(g^{0}_{sol}, T)$
[b] $\Delta G^{TC}_{therm}(T) = G^{+}_{therm}(g^{+}_{gas}, T) - G^{0}_{therm}(g^{0}_{gas}, T)$

Table S7: The energy contribution to GSOP from the geometry changes caused by solvation for neutral and oxidised molecules.

Name	$\Delta E^{0}_{gas\rightarrow sol}$ [eV][a]	$\Delta E^{+}_{gas\rightarrow sol}$ [eV][b]
NDI-44	0.02	-0.03
NDI-54	0.02	-0.04
NDI-55	0.02	-0.02
NDI-555	0.02	0.00
NDI-5555	0.02	0.02
NDI-58	0.02	-0.03
NDI-59	0.02	-0.03
NDI-66	0.02	0.00
NDI-77	0.02	0.02
PDI	0.02	-0.02
PDI-4444	0.02	-0.01
PDI-55c	0.03	0.00
PDI-55t	0.02	0.01
PDI-0000	0.02	-0.04

[a] $\Delta E^{0}_{gas\rightarrow sol} = E^{0}_{gas}(g^{0}_{sol}) - E^{0}_{gas}(g^{0}_{gas})$
[b] $\Delta E^{+}_{gas\rightarrow sol} = E^{0}_{gas}(g^{+}_{sol}) - E^{0}_{gas}(g^{+}_{gas})$

Table S8: The contributions to the electronic energy coming from the relaxation to the oxidized geometry in solution and gas phases

Name	$\Delta E^{sol}_{0\rightarrow +}$ [eV][a]	$\Delta E^{gas}_{0\rightarrow +}$ [eV][b]
NDI-44	0.16	0.21
NDI-54	0.11	0.16
NDI-55	0.10	0.14
NDI-555	0.25	0.26
NDI-5555	0.36	0.36
NDI-58	0.07	0.12
NDI-59	0.07	0.12
NDI-66	0.13	0.15
NDI-77	0.07	0.07
PDI	0.04	0.08
PDI-4444	0.10	0.14
PDI-55c	0.09	0.12
PDI-55t	0.15	0.16
PDI-0000	0.05	0.10

[a] $\Delta E^{sol}_{0\rightarrow +} = E^{0}_{gas}(g^{+}_{sol}) - E^{0}_{gas}(g^{0}_{sol})$
[b] $\Delta E^{gas}_{0\rightarrow +} = E^{0}_{gas}(g^{+}_{gas}) - E^{0}_{gas}(g^{0}_{gas})$

S6 Suitable dyes for the trial system containing the Ru-based water oxidation catalyst designed by Duan et al.[S1] and TiO_2 anode.

Table S9: Calculated values for GOSP [V], lowest most intense excitation λ_{max} [eV] and oscillation strength (osc.str) values for all the dyes with suitable GSOP for the trial system. The left column gives dyes that also fulfil the ESOP criterion. We hereby account for computational errors - underestimation of 0.3 V for GSOP and overestimation of 0.4 eV for λ_{max}.

Num.	Name	GSOP	λ_{max}	osc.str.	Num.	Name	GSOP	λ_{max}	osc.str.
1	PTI1-5	6.00	2.90	0.66	67	PDI	5.89	2.43	0.77
2	PTI1-144	5.88	2.67	0.58	68	PTI1-1	6.46	2.90	0.70
3	PTI1-444	5.81	2.67	0.56	69	PTI1-4	6.49	2.90	0.69
4	PTI1-544	5.91	2.67	0.55	70	PTI1	6.31	2.88	0.67
5	PTI2-4	6.03	2.70	0.55	71	PTI2	6.39	2.89	0.67
6	PTI2-44	5.82	2.64	0.54	72	PTI1-14	6.10	2.72	0.58
7	PTI1-54	6.02	2.72	0.53	73	PTI1-44	6.08	2.72	0.56
8	NDI-125	5.78	2.57	0.45	74	PTI1-11	6.00	2.64	0.44
9	NDI-225	5.84	2.49	0.44	75	NDI-224	5.98	2.48	0.41
10	PTI1-15	5.73	2.49	0.44	76	NDI-422	6.20	2.57	0.40
11	NDI-4622	5.66	2.38	0.43	77	NDI-421	6.11	2.61	0.40
12	PTI1-45	5.67	2.49	0.42	78	NDI-22	6.01	2.43	0.40
13	PTI2-5	5.65	2.47	0.42	79	PTI1-12	6.01	2.54	0.40

14	PTI2-1	5.93	2.62	0.42	80	NDI-121	6.07	2.45	0.39
15	PTI1-55	5.72	2.49	0.42	81	PTI1-52	6.02	2.54	0.39
16	PTI1-41	5.97	2.64	0.42	82	PTI1-42	5.97	2.54	0.39
17	PTI1-51	5.97	2.64	0.40	83	PTI2-2	5.94	2.51	0.38
18	NDI-5422	5.76	2.54	0.39	84	PTI1-22	5.94	2.51	0.37
19	NDI-115	5.67	2.63	0.38	85	NDI-124	5.97	2.52	0.36
20	NDI-5421	5.70	2.59	0.37	86	NDI-6122	5.66	2.19	0.34
21	NDI-426	5.73	2.65	0.37	87	NDI-4224	6.01	2.58	0.34
22	NDI-215	5.81	2.58	0.36	88	NDI-1222	5.83	2.22	0.34
23	NDI-425	5.83	2.72	0.36	89	NDI-412	6.05	2.62	0.34
24	NDI-462	5.73	2.60	0.35	90	NDI-122	5.85	2.32	0.34
25	NDI-5442	5.77	2.73	0.34	91	NDI-411	6.03	2.66	0.33
26	NDI-4446	5.69	2.66	0.33	92	NDI-12	5.96	2.49	0.33
27	NDI-5441	5.70	2.73	0.33	93	NDI-612	5.68	2.32	0.32
28	PTI1-21	5.89	2.58	0.33	94	NDI-4422	6.08	2.59	0.32
29	NDI-461	5.70	2.65	0.33	95	NDI-4412	6.01	2.61	0.32
30	NDI-452	5.85	2.68	0.32	96	NDI-111	5.95	2.48	0.31
31	NDI-214	5.90	2.55	0.32	97	NDI-4114	5.95	2.58	0.31
32	NDI-145	5.75	2.76	0.32	98	NDI-1122	5.72	2.16	0.31
33	NDI-4411	5.92	2.57	0.31	99	NDI-1212	5.83	2.25	0.30
34	NDI-451	5.82	2.73	0.31	100	NDI-1112	5.74	2.24	0.30

35	NDI-524	5.70	2.59	0.30
36	NDI-4621	5.68	2.38	0.30
37	NDI-4642	5.70	2.52	0.30
38	NDI-5444	5.70	2.93	0.30
39	NDI-62	5.65	2.47	0.30
40	NDI-445	5.75	2.87	0.29
41	NDI-5424	5.71	2.69	0.29
42	NDI-114	5.92	2.58	0.29
43	NDI-544	5.66	2.79	0.29
44	NDI-5412	5.67	2.56	0.29
45	NDI-44	6.17	2.87	0.28
46	NDI-52	5.74	2.54	0.28
47	NDI-11	5.89	2.54	0.28
48	NDI-151	5.73	2.51	0.28
49	NDI-444	6.20	3.10	0.27
50	NDI-245	5.92	2.67	0.27
51	NDI-414	6.02	2.82	0.27
52	NDI-4444	6.30	3.21	0.26
53	NDI-454	5.76	2.90	0.26
54	NDI-254	5.70	2.58	0.26
55	NDI-54	5.73	2.73	0.26
101	NDI-2222	5.84	2.10	0.30
102	NDI-4124	6.03	2.59	0.30
103	NDI-4122	5.86	2.32	0.29
104	NDI-4112	5.77	2.34	0.28
105	NDI-222	5.90	2.21	0.27
106	NDI-4	6.62	3.19	0.27
107	NDI-424	6.07	2.70	0.27
108	NDI-112	5.80	2.31	0.26
109	NDI-42	6.04	2.60	0.25
110	NDI-141	6.14	2.60	0.25
111	NDI-4212	5.84	2.30	0.25
112	NDI-212	5.86	2.29	0.24
113	NDI-4222	5.88	2.28	0.23
114	NDI-2	6.23	2.68	0.21
115	NDI-142	6.14	2.36	0.21
116	NDI-1	6.34	2.87	0.21
117	NDI-442	6.22	2.71	0.20
118	NDI-4442	6.12	2.75	0.19

56	NDI-41	6.01	2.68	0.26
57	NDI-262	5.67	2.37	0.25
58	NDI-4441	5.99	2.77	0.24
59	NDI-4262	5.70	2.37	0.24
60	NDI-441	5.99	2.81	0.24
61	NDI-6	5.87	2.81	0.23
62	NDI-4142	5.76	2.44	0.22
63	NDI-5	6.05	2.98	0.21
64	NDI-5242	5.67	2.43	0.21
65	NDI-252	5.72	2.38	0.21
66	NDI-4611	5.74	2.41	0.18

References

(S1) Duan, L.; Bozoglian, F.; Mandal, S.; Stewart, B.; Privalov, T.; Llobet, A.; Sun, L. A molecular ruthenium catalyst with water-oxidation activity comparable to that of photosystem II. *Nat. Chem.* **2012**, *4*, 418–423.

(S2) Röger, C.; Würthner, F. Core-Tetrasubstituted Naphthalene Diimides: Synthesis, Optical Properties, and Redox Characteristics. *J. Org. Chem.* **2007**, *72*, 8070–8075.

(S3) Thalacker, C.; Röger, C.; Würthner, F. Synthesis and Optical and Redox Properties of Core-Substituted Naphthalene Diimide Dyes. *J. Org. Chem.* **2006**, *71*, 8098–8105.

(S4) Kishore, R. S. K.; Kel, O.; Banerji, N.; Emery, D.; Bollot, G.; Mareda, J.; Gomez-Casado, A.; Jonkheijm, P.; Huskens, J.; Maroni, P.; Borkovec, M.; Vauthey, E.; Sakai, N.; Matile, S. Ordered and Oriented Supramolecular n/p-Heterojunction Surface Architectures: Completion of the Primary Color Collection. *J. Am. Chem. Soc.* **2009**, *131*, 11106–11116.

(S5) Bhosale, R.; Kishore, R. S.; Ravikumar, V.; Kel, O.; Vauthey, E.; Sakai, N.; Matile, S. Zipper assembly of SHJ photosystems: Focus on red naphthalenediimides, optoelectronic finetuning and topological matching. *Chem. Sci.* **2010**, *1*, 357–368.

(S6) Bhosale, S. V.; Kalyankar, M. B.; Bhosale, S. V.; Langford, S. J.; Reid, E. F.; Hogan, C. F. The synthesis of novel core-substituted naphthalene diimides via Suzuki cross-coupling and their properties. *New J. Chem.* **2009**, *33*, 2409–2413.

(S7) Jones, B. A.; Facchetti, A.; Wasielewski, M. R.; Marks, T. J. Tuning Orbital Energetics in Arylene Diimide Semiconductors. Materials Design for Ambient Stability of n-Type Charge Transport. *J. Am. Chem. Soc.* **2007**, *129*, 15259–15278.

(S8) Leowanawat, P.; Nowak-Król, A.; Würthner, F. Tetramethoxy-bay-substituted pery-

lene bisimides by copper-mediated cross-coupling. *Org. Chem. Front.* **2016**, *3*, 537–544.

(S9) Ahrens, M. J.; Tauber, M. J.; Wasielewski, M. R. Bis(n-octylamino)perylene-3,4:9,10-bis(dicarboximide)s and their radical cations: Synthesis, electrochemistry, and ENDOR spectroscopy. *J. Org. Chem.* **2006**, *71*, 2107–2114.

(S10) Connelly, N. G.; Geiger, W. E. Chemical redox agents for organometallic chemistry. *Chem. Rev.* **1996**, *96*, 877–910.

(S11) Cardona, C. M.; Li, W.; Kaifer, A. E.; Stockdale, D.; Bazan, G. C. Electrochemical considerations for determining absolute frontier orbital energy levels of conjugated polymers for solar cell applications. *Adv. Mater.* **2011**, *23*, 2367–2371.

(S12) Bard, A. J.; Faulkner, L. R. *Physics (College. Park. Md).*, 2nd ed.; John Wiley & Sons, Inc: New York, 2001.

(S13) Isse, A. A.; Gennaro, A. Absolute Potential of the Standard Hydrogen Electrode and the Problem of Interconversion of Potentials in Different Solvents. *J. Phys. Chem. B* **2010**, *114*, 7894–7899.

(S14) Marenich, A. V.; Ho, J.; Coote, M. L.; Cramer, C. J.; Truhlar, D. G. Computational electrochemistry: Prediction of liquid-phase reduction potentials. *Phys. Chem. Chem. Phys.* **2014**, *16*, 15068–15106.

(S15) Würthner, F. Perylene bisimide dyes as versatile building blocks for functional supramolecular architectures. *Chem. Commun.* **2004**, *4*, 1564–1579.
